# Molecular markers demonstrate diagnostic and prognostic value in the evaluation of myelodysplastic syndromes in cytopenia patients

**DOI:** 10.1038/s41408-022-00612-w

**Published:** 2022-01-25

**Authors:** Rong He, Jonathan Chiou, Allison Chiou, Dong Chen, Constance P. Chen, Caroline Spethman, Kurt R. Bessonen, Jennifer L. Oliveira, Phuong L. Nguyen, Kaaren K. Reichard, James D. Hoyer, Simon D. Althoff, Dana J. Roh, Mechelle A. Miller, Ji Yuan, Horatiu Olteanu, Kebede Begna, Ayalew Tefferi, Hassan Alkhateeb, Mrinal M. Patnaik, Mark R. Litzow, Aref Al-Kali, David S. Viswanatha

**Affiliations:** 1 Division of Hematopathology, Mayo Clinic College of Medicine, Rochester, MN USA; 2 Dartmouth College, Hanover, NH USA; 3 Wellesley College, Wellesley, MA USA; 4 College of Science, University of Notre Dame, Notre Dame, IN USA; 5 School of Medicine, University of Nebraska, Omaha, NE USA; 6 Division of Hematology, Mayo Clinic College of Medicine, Rochester, MN USA

**Keywords:** Risk factors, Myelodysplastic syndrome

**Dear Editor**,

Myelodysplastic syndromes (MDS) are clonal hematopoietic stem cell disorders characterized by cytopenia(s), morphologic dysplasia, ineffective hematopoiesis, recurrent genetic abnormalities, and a variable risk of progression to acute myeloid leukemia. MDS diagnosis is primarily based on the morphologic evaluation for myelodysplasia and the chromosome abnormalities defined by the World Health Organization (WHO) as presumptive evidence for MDS. In recent years, the advent of next-generation sequencing (NGS) has greatly improved our understanding of the genetic basis of MDS and related myeloid disorders. In contrast to cytogenetic abnormalities present in about 50% of MDS patients, somatic mutations are detected in 80–90% of cases, involving pathways of epigenetic modification, RNA splicing, transcription, signal transduction, and DNA repair [1, 2]. Based on a frequent association with ring sideroblasts, *SF3B1* mutations were incorporated into the MDS diagnostic criteria with the 2017 WHO update [3].

The diagnosis of MDS can be challenging in cases lacking diagnostic morphologic features of myelodysplasia, whose accurate evaluation relies heavily on adequately sampled, well-prepared bone marrow aspirate smears, which may not be consistently attainable due to various pathological/technical constraints. Pathologist interobserver variability in myelodysplasia assessment is also well documented [4]. Although the WHO-defined cytogenetic abnormalities and *SF3B1* mutation criteria aid in MDS diagnosis, they only encompass a subset of genetic abnormalities in MDS. Given the high prevalence of somatic mutations in MDS and increasing clinical use of NGS, molecular markers naturally emerge as potential candidates for further diagnostic refinement. However, somatic mutations are not unique to MDS. They are also present in clonal hematopoiesis of indeterminate potential (CHIP), a pre-malignant age-related condition in individuals without cytopenias or hematologic malignancy that confers a 0.5–1% per-year risk of neoplastic progression. Genes most commonly mutated in CHIP are the epigenetic modifiers *DNMT3A*, *TET2*, and *ASXL1* (DTA mutations). As a continuum from CHIP, somatic mutations also occur in clonal cytopenia of undetermined significance (CCUS), a MDS precursor state with unexplained cytopenia and subtle morphologic changes suspicious but not diagnostic for MDS per WHO diagnostic criteria [5, 6]. As MDS is shaped by recursive rounds of clonal evolution, it is not surprising to see some genetic overlap between MDS and its precursor states. However, distinctive genetic features of well-defined MDS may shed light on the identification of molecular markers of diagnostic and prognostic value, particularly in CCUS. In this study, we retrospectively reviewed our institutional experience of a targeted NGS panel in the evaluation of cytopenic patients, aiming to identify unique molecular markers that would help refine MDS diagnosis and improve CCUS prognostication.

The study was approved by the Institutional Review Board of Mayo Clinic. Among consecutive, unexplained cytopenia cases submitted for a myeloid neoplasm (MN)-targeted, 35-gene NGS testing between 2015 and 2017 for the evaluation of MDS, 190 cases fulfilled the diagnostic criteria of MDS, and 116 showed no diagnostic morphologic/cytogenetic/molecular (*SF3B1*) features meeting the 2017 WHO diagnostic criteria of MDS or other MNs (noMN). Baseline characteristics of the study cohort are presented in Table 1. In comparison to noMN, MDS patients showed older age, more frequent male gender, lower hemoglobin (Hb) and absolute neutrophil count (ANC) (*p* < 0.05), but similar platelet count. Chromosome analysis was performed on 184/190 MDS and 115/116 noMN with abnormalities observed in 52.7% and 9.6% (*p* < 0.00001), respectively (Supplementary Table 1).

**Table 1:** Demographic, clinical, and genetic features of 190 MDS and 116 noMN cases.

	MDS		noMN	*p* value
Number of cases (*n*)	MDS-SLD	8		
	MDS-RS	28		
	MDS-MLD	69		
	MDS-EB1	31		
	MDS-EB2	34		
	MDS-U	6		
	MDS-T	12		
	MDS-idel(5q)	2		
	total	190	116	
Age, mean (SD, range), years	71.3 (9.6, 20–90)		61.6 (16.6, 14–87)	<0.00001
Gender (% male, male/female)	70.5%, 134/56		58.6%, 68/48	0.04
Hb, mean (SD, range), g/dL	9.4 (1.8, 5.2–14.6)		10.3 (2.3, 5.7–15.8)	0.0002
ANC, mean (SD, range), ×10^9^/L	1.9 (0.03–16.5)		2.5 (2.0, 0.06–8.9)	0.02
Platelet count, mean (SD, range), ×10^9^/L	123 (120, 7–632)		133 (93, 7–510)	0.49
Mutated cases (%, *n*)	84.7% (161/190)		22.4% (26/116)	<0.00001
Number of mutations/case, mean (SD, range)	2.1 (0.6, 0–8)		0.3 (0.7, 0–3)	<0.00001
Mutational VAF^a^, mean (SD, range)	40% (18.9%, 5.6–100%)		32.5% (22.5%, 6.2–87%)	0.07
Abnormal karyotype (%, *n*)	52.7% (97/184)		9.6% (11/115)	<0.00001

*MDS* myelodysplastic syndrome, *noMN* cytopenia cases not meeting the diagnostic criteria of MDS or other myeloid neoplasms, *MDS-SLD* MDS with single lineage dysplasia, *MDS-RS* MDS with ringed sideroblasts, *MDS-idel(5q)* MDS with isolated del(5q), *MDS-MLD* MDS with multilineage dysplasia, *MDS-EB* MDS with excess blasts, *MDS-U* MDS-unclassifiable, and *MDS-T* therapy-related MDS, *SD* standard deviation, *VAF* variant allele fraction, *Hb* hemoglobin, *ANC* absolute neutrophil count.

^a^Highest mutational VAF of the case if multiple mutations present.

In MDS patients, pathogenic/likely pathogenic mutations were identified in 84.7% (161/190) with a mean number of 2.1 mutations and mean variant allele fraction (VAF) of 40% (Table 1). The mutational landscape is depicted in Fig. 1A, with the most common mutations occurring in *ASXL1* (26.8%), *TET2* (20%), *SRSF2* (17.9%), *SF3B1* (17.4%), *RUNX1* (15.3%), *U2AF1* (14.7%), *TP53* (12.1%), and *DNMT3A* (11.6%). Mutations occurred in a full spectrum of pathways, including epigenetic modifiers (EM, 58.9%), splicing factors (SF, 50.5%), transcription factors (TF, 20.5%), tumor suppressors (TS, 15.3%) and signaling and kinase pathways (SKP, 11.1%).

**Fig. 1:**
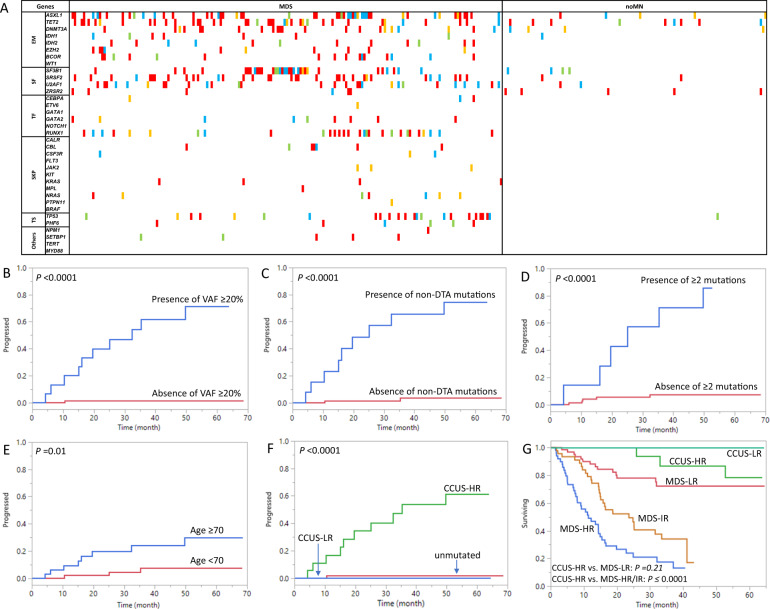
Mutational landscape of the study cohort and Kaplan-Meier analysis in the noMN patients. **A** Mutational landscape of the 190 MDS and 116 noMN patients. Mutational VAF is color coded: green, <10%; orange: 10–19.9%; blue: 20–29.9%; red, ≥ 30%. If multiple mutations are present in a case, the highest VAF is shown. In MDS, mutations occurred most frequently in *ASXL1, TET2, SRSF2, SF3B1, RUNX1, U2AF1, TP53*, and *DNMT3A*, all involving ≥10% of MDS cases. Less frequent mutations were seen in *BCOR, IDH2, EZH2, GATA2, CBL, IDH1, NRAS, PHF6, ZRSR2, SETBP1, CEBPA, JAK2, KRAS, ETV6, MPL, NPM1*, and *PTPN11*, in descending order of frequency (8.4–0.5%). In noMN, mutations occurred in *TET2* (6.9%), *ASXL, ZRSR2* (each 4.3%), *DNMT3A, SF3B1* (each 3.4%), *SRSF2, U2AF1* (each 1.7%), and *TP53* and *IDH1* (each 0.9%). **B**–**G** In the 116 noMN patients, the median time to MN progression was 32.4 months in patients with VAF ≥ 20% (*n* = 16) vs. not reached in patients without VAF ≥ 20% (unmutated or mutational VAF < 20%, *n* = 100), log rank *p* < 0.0001 (**B**); 25.2 months in patients with non-DTA mutations (*n* = 14) vs. not reached in patients without a non-DTA mutation (unmutated or with DTA-only mutations, *n* = 102), log rank *p* < 0.0001 (**C**); 25.2 months in patients with ≥2 mutations (*n* = 7) vs. not reached in patients without ≥2 mutations (unmutated or with one mutation, *n* = 109), log rank *p* < 0.0001 (**D**); Age ≥70 years (*n* = 45) was associated with MN progression in comparison to age <70 years (*n* = 71), median not reached in both, *p* = 0.01 (**E**). CCUS with high-risk markers (CCUS-HR, *n* = 19) showed a median time to progression of 35.2 months vs. not reached in the CCUS low-risk group (CCUS-LR, *n* = 7) and unmutated noMN group (*n* = 90), log rank *p* < 0.0001 (**F**). The OS of CCUS-HR (*n* = 19) was similar to that of low-risk MDS (MDS-LR, *n* = 72, median not reached in both, log rank *p* = 0.21) but superior compared to high-risk MDS (MDS-HR, median 11.4 months, *n* = 57, log rank *p* < 0.0001) and intermediate-risk MDS (MDS-IR, median 23.6 months, *n* = 55, log rank *p* = 0.0001) (**G**). *EM* epigenetic modifiers, *SF* splicing factors, *TF* transcription factors, *SKP* signaling and kinase pathway factors, *TS* tumor suppressors, *MN* myeloid neoplasm, *VAF* variant allele fraction, *CCUS* clonal hematopoiesis of undetermined significance.

In the 116 noMN patients, in comparison to MDS, pathogenic/likely pathogenic mutations were less frequent (26/116, 22.4%, *p* < 0.00001), with a lower number of mutations per case (mean 0.3, *p* < 0.00001) yet similar VAF (mean 32.5%, *p* = 0.07) (Table 1). Mutations were limited to the EM (15.6%, *TET2/ASXL1/DNMT3A/IDH1*), SF (10.3%, *ZRSR2/SF3B1/SRSF2/U2AF1*), and TS (0.9%, *TP53*) pathways, with no SKP or TF mutations observed (Fig. 1A). The clinical features of the 26 mutated noMN (CCUS) patients are described in Supplementary Table 2. 21 of 116 noMN patients received erythropoiesis-stimulating agents (6.9%), immune suppressants (10.3%), or hypomethylating agent (0.9%).

The diagnostic value of various molecular markers for MDS was analyzed (Supplementary Table 3). The presence of mutation(s) (≥1 mutation) exhibited the highest sensitivity of 84.7% and best negative predictive value (NPV) of 75.6%, with a specificity of 77.6% and positive predictive value (PPV) of 86.1%. The presence of a mutational VAF ≥ 20%, a mutation in a gene other than DTA (non-DTA mutations), or ≥2 mutations were highly predictive of MDS and all demonstrated an excellent PPV > 90%. Raising the diagnostic cut-off to ≥3 mutations or adding VAF ≥ 10–30% to ≥2 or ≥3 mutations showed minimal improvement in PPV. The presence of TF, SKP, or ≥4 mutations was restricted in MDS and each exhibited a PPV and specificity of 100%, with correspondingly lower NPV and sensitivity. The best overall diagnostic performance was observed in non-DTA mutations (Youden index 0.66). Conversely, the worst predictor was the presence of DTA-only mutations (Youden index −0.05).

We then evaluated the prognostic value of molecular markers in MN progression in the 116 noMN patients. Between 26 CCUS patients harboring 1–3 mutations and 90 unmutated noMN patients, no significant difference was observed in overall survival (OS) (median, both not reached, *p* = 0.51) over a median follow-up time of 25.9 months (range 0.3–68.5). Eleven cases progressed to MN, including 10/26 CCUS and 1/90 unmutated patient harboring trisomy 8 (*p* < 0.0001, Supplementary Table 2). In Kaplan–Meier analysis, the three diagnostic markers highly predictive of MDS (i.e., VAF ≥ 20%, non-DTA mutations, and ≥2 mutations) were all significantly associated with MN progression (median time to progression, 32.4 months vs. not reached, *p* < 0.0001; 25.2 months vs. not reached, *p* < 0.0001; 25.2 months vs. not reached, *p* < 0.0001, respectively, Fig. 1B–D). Age ≥70 years (Fig. 1E) and male gender were also associated with MN progression (both not reached, *p* = 0.01 and *p* = 0.04, respectively). Univariate Cox proportional hazard regression analysis showed a MN-progression hazard ratio (HR) of 59.5, 35.7, 16, and 4.8 for VAF ≥ 20% (*p* = 0.0001), non-DTA mutations (*p* < 0.0001), ≥2 mutations (*p* < 0.0001), and age ≥70 (*p* = 0.02), respectively (Supplementary Table 4). Gender, Hb, ANC, platelet count, and cytogenetic abnormalities showed no significant impact. Multivariate analysis demonstrated VAF ≥ 20% and non-DTA mutations as independent high-risk progression markers in noMN, exhibiting a HR of 44.8 (*p* = 0.01) and 13.1 (*p* = 0.046), respectively.

The two independent high-risk progression markers were further validated in the 26 CCUS patients. High-risk CCUS (CCUS-HR) bearing at least one of these markers showed significantly higher risk of MN progression than low-risk CCUS (CCUS-LR) or unmutated noMN cases (median 35.2 month vs. not reached in the latter two groups, *p* < 0.0001, Fig. 1F), however no difference in OS was observed (*p* = 0.59). The OS of CCUS-HR was similar to that of low-risk MDS (MDS-LR, IPSS-R low/very low, median both not reached, *p* = 0.21) but superior compared to high-risk MDS (MDS-HR, IPSS-R high/very high risk, 11.4 months, *p* < 0.0001) and intermediate-risk MDS (MDS-IR, IPSS-R intermediate risk, 23.6 months, *p* = 0.0001) (Fig. 1G). Clinical and hematologic features were also comparable between CCUS-HR and MDS-LR patients (*p* > 0.05, Supplemental Table 5).

Increased clinical use of NGS results in a higher prevalence of CCUS, a common diagnostic and clinical management conundrum. CCUS patients can be as symptomatic as MDS patients but may not qualify for MDS therapy or clinical trials. Concurrently, accumulating molecular and clinical data provide unprecedented opportunities for the discovery of novel markers for enhancing patient care in CCUS and MDS. In our retrospective evaluation of 306 patients with unexplained cytopenia(s) and NGS testing, three molecular markers were highly predictive of MDS: VAF ≥ 20%, non-DTA mutations, and ≥2 mutations. Multivariable analysis confirmed the first two as independent progression risk markers in noMN, which was further validated in CCUS. CCUS-HR patients also showed significant clinical/hematologic overlap with MDS-LR patients. These findings support that high-risk molecular markers of VAF ≥ 20% and non-DTA mutations may serve as presumptive evidence for MDS in CCUS, akin to the application of WHO-defined cytogenetic abnormalities for MDS diagnosis.

The strength of the study includes a large cohort of well-characterized MDS and noMN cases with clinical follow-up, identification of MDS diagnostic markers, followed by their prognostic assessment and validation in the noMN cohort. The limitations include a relatively small CCUS cohort within noMN and the relatively short follow-up time (25.9 months). Despite these limitations, we demonstrate evident diagnostic and prognostic value of molecular markers in MDS evaluation of cytopenic patients. The results presented here comport with and expand the existing literature illustrating clinical value of similar molecular markers in MDS diagnosis and CCUS prognostication, and strengthens the uniform call for evidence-based risk stratification of CCUS and diagnosis refinement of MDS to advance patient care [7–15].

## Supplementary information

Supplementary materials
