# Supplementary material for: Molecular markers demonstrate diagnostic and prognostic value in the evaluation of myelodysplastic syndromes in cytopenia patients
Source: Blood Cancer J. 2022 Jan 25;12(1):12. doi: 10.1038/s41408-022-00612-w (PMC8789920; doi:10.1038/s41408-022-00612-w)
Supplement: Supplementary file 1 — Supplementary materials [file 41408_2022_612_MOESM1_ESM.docx]

**Supplementary Information**

Table of contents

Methods page 2

Supplementary table 1 page 4

Supplementary table 2 page 17

Supplementary table 3 page 20

Supplementary table 4 page 21

Supplementary table 5 page 22

**Methods**

**Case selection and data collection**

Following Mayo Clinic Institutional Review Board approval, we retrospectively screened Mayo Clinic cases that had a targeted 35-gene OncoHeme NGS panel performed for the evaluation of MDS in patients with unexplained cytopenias. Cytopenia was defined as hemoglobin (Hb) < 13g/dL in men, < 12g/dL in women, absolute neutrophil count (ANC) < 1.8 x 10^9^/L, or platelets < 150 x 10^9^/L. Cases diagnosed with MDS, as well as those not meeting the diagnostic criteria of MDS or other myeloid neoplasms (per 2017 WHO diagnostic criteria), were included in the study. Nine cases were excluded from further analysis, including three cases of aplastic anemia, three congenital cytopenia, one myeloproliferative neoplasm, one VEXAS syndrome, and one inadequate bone marrow specimen. The bone marrow pathologic diagnoses were verified by at least two hematopathologists. Clinical course and laboratory and clinical findings were collected by chart review.

**Cytogenetic analysis**

Fresh bone marrow aspirate samples were cultured and harvested following standard cytogenetic methods and chromosome preparations were stained using GTL banding with trypsin and Leishman stain.  A total of 20 metaphases were analyzed and reviewed for each sample when available. Fluorescence in situ hybridization (FISH) analysis was performed using commercial or laboratory-developed probes. A total of 500 nuclei were scored for dual-color, dual-fusion (D-FISH) probes designed to identify translocations or inversions, and 200 nuclei for probes detecting copy number changes (e.g., +8), deletions (e.g., 5q-), or break-apart probes detecting gene disruptions. FISH analyses were independently evaluated by two technologists. MDS FISH panel targets included inv(3) RPN1/MECOM fusion, -5q, -5, -7q, -7, +8, -13q, -20q, -20q, -11q, -17p, and -17.

**Next-Generation Sequencing (NGS) analysis**

NGS testing was performed using a targeted, myeloid neoplasm-focusing OncoHeme panel (NGSHM), which interrogates 35 genes, including epigenetic modifiers (EM) *ASXL1, BCOR, DNMT3A, TET2, IDH1, IDH2, EZH2, and WT1;* splicing factors (SF) *SF3B1, SRSF2, U2AF1, and ZRSR2;* transcription factors (TF) *CEBPA, ETV6, GATA1, GATA2, NOTCH1, RUNX1;* signaling and kinase pathway factors (SKP) *BRAF,* *CALR, CBL, CSF3R, FLT3, JAK2, KIT, KRAS, MPL, MYD88, NRAS, PTPN11;* tumor suppressors (TS) *TP53* and *PHF6*; molecular chaperone *NPM1*; telomerase reverse transcriptase *TERT;* and the functionally poorly defined *SETBP1.* DNA was extracted from bone marrow or peripheral blood using Qiagen EZI (Qiagen, Germantown, MD). NGS was performed using 200ng sheared DNA with a custom hybridization-capture reagent (SureSelect^XT^, Agilent, Santa Clara, CA) and sequenced on the MiSeq or HiSeq platform (Illumina, San Diego, CA). Genetic variants were curated and annotated in the Mayo Clinic Molecular Hematopathology Laboratory based on the American College of Medical Genetics and Genomics (ACMG) five-tier system with minor modifications for hematologic neoplasms [1, 2]. The analytic sensitivity of the assay is 5% with a minimum 250X depth of coverage. More than 95% of tested regions had > 1000X depth of coverage in the clinical assay. Pathogenic and likely pathogenic mutations were included in the study analysis.

**Statistical analysis**

Numerical variables were presented by mean, standard deviation (SD) and range; categorical variables were described by count and percentage in each category. Categorical variables and numerical variables among the MDS and noMN groups were compared by Fisher’s exact test and t-test, respectively. Overall survival (OS) was defined as the time from the date of the NGS testing to the date of death (for deceased patients) or last follow-up (for censored patients). Time to progression was defined as the time from the date of NGS testing to the date of myeloid neoplasm progression (for progressed patients) or last follow-up (for censored patients). Kaplan-Meier survival curve (log-rank test) and Cox proportional hazards regression model (both univariate and multivariate analyses) were used to identify the potential clinical impact of different variables and were performed by using JMP Pro software version 14 (SAS Institute Inc., Cary, NC). The main end point in this study for the noMN group was progression to myeloid neoplasm. Statistical significance was based on a *p* value < 0.05.

References

1. Richards S, Aziz N, Bale S, Bick D, Das S, Gastier-Foster J, et al. Standards and guidelines for the interpretation of sequence variants: a joint consensus recommendation of the American College of Medical Genetics and Genomics and the Association for Molecular Pathology. Genet Med. 2015;17(5):405-24.

2. Mehta N, He R, Viswanatha DS. Internal Standardization of the Interpretation and Reporting of Sequence Variants in Hematologic Neoplasms. Mol Diagn Ther. 2021;25(4):517-26.

Supplementary Table 1. Demographic, clinical, and genetic findings of 190 MDS and 116 noMN cases.

| **Case number** | **Age** | **Gender** | **Disease category** | **Mutation summary** | **Cytogenetic results** |
| --- | --- | --- | --- | --- | --- |
| 1 | 56 | F | 1 | None | 45,X?c[15]/46,XX[5] |
| 2 | 69 | M | 1 | 1. *ASXL1*: c.1934dup; p.Gly646Trpfs*12; 31.2%; 2. *GATA2*: c.1165_1173del; p.Lys389_Glu391del; 35.6%; 3. *ZRSR2*: c.883C>T; p.Arg295*; 79.4% | 46,XY[20] |
| 3 | 67 | F | 1 | 1. *ASXL1*: c.1934dup;p.Gly646Trpfs*12. 38.1%. 2. *SRSF2*: c.284C>T;p.Pro95Leu; 43.8; 3. *TET2*: c.3646C>T;p.Arg1216*; 45.9% | 46,XX[20] |
| 4 | 64 | F | 1 | None | 46,XX[20] |
| 5 | 56 | M | 1 | None | 46,XY,del(13)(q12q14)[19]/46,XY[1] |
| 6 | 80 | F | 1 | 1. *TET2*: c.3718_3719del; p.Leu1240Glyfs*2. 40; | 46,XX[20] |
| 7 | 79 | M | 1 | 1. *ASXL1*: c.1934dup; p.Gly646Trpfs*12; 40.7%; 2. *TET2*: c.1441C>T; p.Gln481*; 40.5%; 3. *RUNX1*: c.908dup; p.Ser304Glnfs*269; 40.3% | 46,XY[20] |
| 8 | 83 | M | 1 | 1. *TP53*: c.783-1G>A; p.?; 6.4%; 2. *TP53*: c.537T>A; p.His179Gln; 8.9% | 46,XY[20] |
| 9 | 79 | M | 2 | 1. *TET2*: c.285del; p.Lys95Asnfs*18; 34%; 2. *TET2*: c.4138C>T; p.His1380Tyr; 28%; 3. *ZRSR2*: c.827+1G>A, p.?; 53.4% | 46,XY[20] |
| 10 | 82 | F | 2 | 1. *SRSF2*: c.284C>G; p.Pro95Arg; 31.%; | 46,XX[20] |
| 11 | 78 | M | 2 | 1. ASXL1: c.1900_1922del; p.Glu635Argfs*15; 23.4%;  2. *EZH2*: c.1708C>T; p.Gln570*; 45.2%;  3. *IDH1*: c.394C>T; p.Arg132Cys; 29.7%;  4. *NRAS*: c.35G>A; p.Gly12Asp; 39.9%;  5. *RUNX1*: c.643_656del; p.His215Alafs*14; 10.6%;  6. *RUNX1*: c.607C>T; p.Gln203*; 26.5% | 47,XY,+8[2]/46,XY[18] |
| 12 | 66 | M | 2 | 1. *SF3B1*: c.1873C>T; p.Arg625Cys; 40.7%; 2. *TET2*: c.1648C>T; p.Arg550*; 43.6%; 3.*TET2*: c.3732_3733del; p.Tyr1245Leufs*22. 40.4% | 46,XY[20] |
| 13 | 49 | M | 2 | 1. *ASXL1*: c.2755_2819dup; p.Pro941Tyrfs*26; 26.1%;  2. *U2AF1*: c.101C>T; p.Ser34Phe; 36.7% | 46,XY,i(8)(q10)[19]/46,XY[1] |
| 14 | 81 | M | 2 | 1. *ASXL1*: c.1641dup; p.Phe548Leufs*3. 44.9%;  2. *CSF3R*: c.1853C>T; p.Thr618Ile; 22.9%;  3. *EZH2*: c.2016C>G; p.Phe672Leu; 93.1%;  4. *GATA2:* c.416_417del; p.Ser139Cysfs*45; 17.6%;  5. *TET2*: c.4893T>G; p.Tyr1631*; 12.5% | 46,XY[20] |
| 15 | 87 | M | 2 | 1. *BCOR*: c.529_530del; p.Ser177Profs*8; 38.7%; 2. *BCOR*: c.3649C>T; p.Arg1217*; 5.2%; 3. *EZH2*: c.1896del; p.Phe632Leufs*43; 85.3%; 4. *RUNX1*: c.237_249del; p.Trp79*; 26.5% | 46,XY[20] |
| 16 | 65 | M | 2 | 1. *BCOR*: c.2319_2323dup; p.Leu775Serfs*13; 65.3%;  2. *DNMT3A*: c.2296A>T; p.Lys766*; 43%;  3. *EZH2*: c.1876G>A; p.Val626Met; 27.1%;  4. *U2AF1*: c.101C>T; p.Ser34Phe; 41.1%. | 46,XY,add(5)(q31)[9]/47,XY,+8[1]/46,XY[10] |
| 17 | 76 | M | 2 | 1. *ASXL1*: c.1934dup; p.Gly646Trpfs*12; 35.7%;  2. *U2AF1*: c.101C>T; p.Ser34Phe; 39.9% | 46,XY,del(20)(q11.2q13.3)[20] |
| 18 | 52 | F | 2 | None | 46,XX[20] |
| 19 | 65 | F | 2 | 1. *IDH2*: c.419G>A; p.Arg140Gln; 47.5%;  2. *SRSF2*: c.281_283dup; p.Arg94dup; 36.6% | 46,XX[20] |
| 20 | 59 | M | 2 | 1. *BCOR*: c.2333del; p.Asp778Valfs*8; 5.9%;  2. *RUNX1*: c.632_645del; p.Val211Alafs*18; 50.8% | 46,XY[20] |
| 21 | 71 | F | 2 | 1. *U2AF1*: c.470A>C; p.Gln157Pro; 26.8% | 46,XX,del(7)(q22q34)[2]/47,XX,+8[2]/46,XX[16] |
| 22 | 63 | M | 2 | None | 46,XY[12] |
| 23 | 56 | F | 2 | None | 46-47,XX,add(3)(q21),t(3;13)(p13;q32),add(5)(q11.2),-6,+8,+11,add(11)(p15)x2,add(16)(q24), -18,i(18)(p10),+r[cp6]/46,XX[14] |
| 24 | 72 | M | 2 | 1. *ASXL1*: c.1268dup; p.Asn423Lysfs*15; 31%;  2. *NRAS*: c.35G>T; p.Gly12Val; 17%;  3. *TET2*: c.3646C>T; p.Arg1216*; 35%;  4. *TET2*: c.5396dup; p.Met1800Aspfs*6; 33.1% | 45,X,-Y[20] |
| 25 | 75 | F | 2 | None | 46,XX[20] |
| 26 | 82 | M | 2 | 1. *U2AF1*: c.470A>G; p.Gln157Arg; 20% | 46,XY[20] |
| 27 | 73 | M | 2 | 1. *ASXL1*: c.1723C>T; p.Gln575*; 38.1%;  2. *BCOR*: c.4428+1G>A; p.?; 60.9%;  3. *CEBPA*: c.625del; p.Gln209Argfs*109; 15.7%;  4. *IDH1*: c.394C>T; p.Arg132Cys; 6.9%;  5. *RUNX1*: c.943_955dup; p.Arg319Hisfs*258; 15.8%; | 46,XY[20] |
| 28 | 74 | F | 2 | None | 46,XX,inv(3)(q21q26.2),add(5)(q11.2)[3]/46,XX[17] |
| 29 | 60 | F | 2 | 1. *ASXL1*: c.2586del; p.Phe862Leufs*5; 9% | 46,XX[20] |
| 30 | 69 | M | 2 | 1. *IDH2*: c.419G>A; p.Arg140Gln; 41.1%;  2. *SRSF2*: c.284_307del; p.Pro95_Arg102del; 29.8% | 46,XY[20] |
| 31 | 82 | F | 2 | None | 46,XX,t(3;11)(q12;q21),del(9)(q13q22),der(11)t(3;11)(q21;q23)[11]/46,XX[5] |
| 32 | 84 | M | 2 | 1. *SETBP1*: c.2608G>A;p.Gly870Ser; 5.6% | 46,XY,del(5)(q13q33)[5]/46,sl,t(Y;14)(p11.2;q11.2)[13]/47,sdl1,+21[1]/46,XY[1] |
| 33 | 82 | M | 2 | 1. *ASXL1*: c.1829dup; p.Ala611Argfs*8; 27% | 46,XY,del(7)(q22)[17]/46,XY[3] |
| 34 | 67 | F | 2 | None | 46,XX,add(1)(p36.1),add(2)(q31),del(5)(q22q31), del(7)(q22q34),add(9)(q22), -20,+mar[4]/46,XX[16] |
| 35 | 59 | M | 2 | 1. *U2AF1*: c.101C>T; p.Ser34Phe; 19.6% | 46,XY,der(6)t(6;11)(p23;q13)[19]/46,XY[1]. |
| 36 | 75 | M | 2 | 1. *RUNX1*: c.319G>A;p.Ala107Thr; 10.6%; 2. *SRSF2*: c.284C>T;p.Pro95Leu; 34.2%; 3. *SRSF2*: c.284C>G;p.Pro95Arg; 10.7%; 4. *TET2*: c.960del;p.Glu320Aspfs*27; 33.7%; 5. *TET2*: c.487_488del;p.Phe163Hisfs*7; 23.5%; 6. *TET2*: c.3501. 2A>T; p.?; 10.4%; 7. *TET2* :3398dup; p.Cys1133Trpfs*9; 10%; 8. *TET2* : 4190C>T; p.Thr1397Ile; 5% | 46,XY[20] |
| 37 | 77 | M | 2 | 1. *IDH1*: c.395G>A; p.Arg132His; 35.9%; 2. *SRSF2*: c.281_283dup; p.Arg94dup; 35.7% | 46,XY[20] |
| 38 | 68 | M | 2 | 1. *ASXL1*: c.1934dup; p.Gly646Trpfs*12; 28.7%; 2. *SRSF2*: c.284C>T; p.Pro95Leu; 42.3%; 3. *TET2*: c.2736_2737delinsTT; p.Gln913*; 40.8%; 4. *TET2*: c.3764del; p.Tyr1255Serfs*11. 46.2% | 47,XY,+8[1]/46,XY[19] |
| 39 | 60 | M | 2 | 1. *BCOR*: c.2265C>G;p.Tyr755*; 8.8%; 2. *PHF6*: c.715C>T;p.His239Tyr; 70.9% | 46,XY,del(20)(q11.2q13.3)[12]/47,idem,+19[8] |
| 40 | 63 | F | 2 | 1. *KRAS*: c.34G>C; p.Gly12Arg; 38.1% | 46,XX[20]. |
| 41 | 62 | F | 2 | None | 46,XX,inv(3)(p25q21),del(5)(q13q33)[18]/46,XX[2] |
| 42 | 77 | M | 2 | 1. *ASXL1*: c.1934dup;p.Gly646Trpfs*12; 32.1%. 2. *IDH1*: c.395G>A;p.Arg132His; 32.4%; 3. *U2AF1*: c.470A>C;p.Gln157Pro; 32% | 46,XY[20] |
| 43 | 80 | M | 2 | 1. *TET2*: c.3893G>A; p.Cys1298Tyr; 44.7%; 2. *U2AF1*: c.467G>A; p.Arg156His; 36.6% | NA |
| 44 | 73 | F | 2 | 1. *IDH2*: c.419G>A; p.Arg140Gln; 29.4%; 2. *SRSF2*: c.284C>G; p.Pro95Arg; 32.5% | 46,XX,+1,der(1;22)(q10;q10)[7]/46,XX,+1,der(1;21)(q10;q10)[3]/46,XX[10] |
| 45 | 66 | F | 2 | None | 46,XX,del(5)(q22q35),del(7)(p13)[6]/46,XX[14] |
| 46 | 59 | M | 2 | 1. *ASXL1*: c.1900_1922del; p.Glu635Argfs*15; 52. | 47,XY,del(1)(p32p36.1),+8,i(17)(q10)[20] |
| 47 | 61 | M | 2 | 1. *DNMT3A*: c.2206C>T; p.Arg736Cys; 89%; 2. *SF3B1*: c.1873C>T; p.Arg625Cys; 42%; 3. *TET2*: c.1504del; p.Ser502Leufs*31; 15%; 4. *TET2*: c.3594+2dup; p.?; 42%; 5. *TET2*: c.4553del; p.Ser1518*; 21.1% | 46,XY[20] |
| 48 | 65 | F | 2 | 1. *TP53*: c.310C>T; p.Gln104*; 17% | 45,XX,psu dic(2;3)(p13;p21)add(2)(q33),add(5)(q11.2)[11]/44,sl,-14,der(15)t(14 ;15)(q12;p13)[3]/46,XX[6] |
| 49 | 68 | M | 2 | 1. *DNMT3A*: c.2599del; p.Val867Tyrfs*14; 17% | 47,XY,+9[7]/46,XY[7]//46,XX[6]. |
| 50 | 72 | M | 2 | None | 46,XY[20]. |
| 51 | 74 | M | 2 | 1. *DNMT3A*: c.1343dup; p.Tyr448*; 39.9%; 2. *SF3B1*: c.2098A>G; p.Lys700Glu; 38.5 | 46,XY[20] |
| 52 | 68 | M | 2 | 1. *CBL*: c.1259G>A; p.Arg420Gln; 89.2%; 2. *SRSF2*: c.284C>G; p.Pro95Arg; 50%; 3. *ZRSR2*: c.827+1G>A; p.?; 100% | 46,XY,add(15)(p11.2),del(20)(q11.2q13.3)[20] |
| 53 | 73 | M | 2 | 1. *U2AF1*: c.101C>A; p.Ser34Tyr; 35% | 46,XY,del(20)(q11.2q13.1)[20] |
| 54 | 47 | M | 2 | 1. *TP53*: c.517G>A; p.Val173Met; 45% | 44-48,XY,-5,-7,add(12)(p11.2),del(12)(p11.2p13),hsr(12)(p11.2),+0-2r ,+0-2mar[cp20 |
| 55 | 74 | M | 2 | 1. *ASXL1*: c.1934dup; p. Gly646Trpfs*12 ; 33.2%;  2. *SRSF2*: c.284C>T; p. Pro95Leu; 35.2%;  3. *TET2*: c.4502_4505del; p. Gln1501Leufs*69; 30.3%;  4. *TET2*: c.5551_5554dup; p. Gln1852Argfs*8 ; 26.6%. | 46,XY[20] |
| 56 | 74 | F | 2 | 1. *IDH2:* c.419G>A; p.Arg140Gln ; 25.4% | 46,XX[20] |
| 57 | 74 | M | 2 | 1. *RUNX1*: c.393_403delinsCAATGACCTCT; p.Arg135Trp; 8.1%;  2. *SRSF2*: c.283_284insGGC; p.Arg94dup; 37.2%;  3. *TET2*: c.1795C>T; p.Gln599*; 45%;  4. *TET2*: c.4546C>T; p.Arg1516*; 44.1% | 46,XY[20]. |
| 58 | 81 | F | 2 | 1. *DNMT3A*: c.1319G>A; p.Trp440*; 41%;  2. *TET2*: c.483dup; p.Asp162Argfs*9; 39%;  3. *TET2*: c.2268dup; p.Leu757Thrfs*12; 14%;  4. *TET2*: c.5618T>C; p.Ile1873Thr; 7.1%;  5. *TP53*: c.818G>A; p.Arg273His; 35% | 45,XX,-5,+mar[6]/43,idem,-4,-7,dic(12;16)(p11.2;p11.2),add(15)(q11.2),add(19)(q13.1)[2]/ 46,XX[12] |
| 59 | 75 | M | 2 | 1. *DNMT3A*: c.1530del; p.Gly511Glufs*140; 38.1%; 2. *SF3B1*: c.2098A>G; p.Lys700Glu; 33% | 46,XY[20] |
| 60 | 68 | M | 2 | 1. *BCOR*: c.760_784del; p.Gly254Hisfs*4. 65.2%;  2. *GATA2*: c.297dup; p. Gly100Argfs*85; 28%;  3. *RUNX1*: c.305_308delinsGGTAGAC; p.Leu102Tyr453delinsArg; 28% | 47,XY,+8[14]/46,XY[6] |
| 61 | 74 | M | 2 | 1. *ASXL1*: c.2113G>T; p.Glu705*; 32.1%; 2. *U2AF1*: c.101C>T; p.Ser34Phe; 29% | 46,XY,del(20)(q11.2q13.3)[14]/46,XY[6] |
| 62 | 73 | M | 2 | None | 46,XY[20] |
| 63 | 54 | M | 2 | 1. *ASXL1*: c.1900_1922del; p.Glu635Argfs*15; 42.1%;  2. *U2AF1*: c.101C>A; p.Ser34Tyr; 36% | 46,XY,del(20)(q11.2q13.3)[20] |
| 64 | 75 | M | 2 | 1. *TET2*: c.4134_4135del; p.Ala1379Serfs*21; 38% | 46,XY[20] |
| 65 | 85 | M | 2 | None | 45,X,-Y,del(11)(q13q23)[17]/46,XY[3]. |
| 66 | 78 | M | 2 | 1. *ASXL1*: c.1934dup; p.Gly646Trpfs*12; 20.1%;  2. *SRSF2*: c.284C>A; p.Pro95His; 22.6% | 47,XY,+8[20] |
| 67 | 76 | M | 2 | 1. *ASXL1*: c.1934dup; p.Gly646Trpfs*12; 32.4%;  2. *EZH2*: c.434dup; p.Ile146Hisfs*2; 63.7%;  3. *TET2*: c.1267A>T; p.Lys423*; 32%;  4. *TET2*: c.2490del; p.Gln831Argfs*10; 34.9% | 47,XY,add(3)(q12),+8[5]/46,XY[15] |
| 68 | 81 | F | 2 | 1. *ASXL1*: c.2197C>T; p.Gln733*; 40.1%;  2. *SRSF2*: c.284C>A; p.Pro95His; 37.8%;  3. *SETBP1*: c.2608G>A; p.Gly870Ser; 9.4% | 46,XX,del(20)(q11.2q13.3)[20] |
| 69 | 60 | M | 2 | None | 47-51,XY,del(11)(q13q23),+19,-20,+1-5mar[cp20] |
| 70 | 54 | F | 2 | None | 46,XX[20] |
| 71 | 82 | M | 2 | None | 45,X,-Y[3]/46,XY[17] |
| 72 | 59 | F | 2 | None | 46,XX[20] |
| 73 | 70 | F | 2 | 1. *TP53*: c.401T>G; p.Phe134Cys; 11.1% | 45,X,-X,add(1)(p36.3),-3,add(5)(q11.2),add(6)(p21),-7,-18,+3 mar[8]/45,XX,-4,add(5)(q11.2),t(9;12)(p13;q24.1),-14,add(20) (q11.2),+mar[3]/46,XX[9] |
| 74 | 73 | M | 2 | 1. *ASXL1*: c.1934dup; p.Gly646Trpfs*12; 11% | 45,X,-Y[5]/46,XY[15] |
| 75 | 69 | M | 2 | 1. *DNMT3A*: c.2645G>A; p.Arg882His; 32.5%;  2. *U2AF1*: c.470A>G; p.Gln157Arg; 5.2% | 47,XY,+8[4]/46,XY[16] |
| 76 | 77 | F | 2 | 1. *ASXL1*: c.1934dup; p.Gly646Trpfs*12; 34.1%;  2. *U2AF1*: c.470A>C; p.Gln157Pro; 39.2% | 46,XX,i(14)(q10)[20] |
| 77 | 78 | F | 3 | 1. *EZH2*: c.1876G>A; p.Val626Met; 11.3%;  2. *SF3B1*: c.2098A>G; p.Lys700Glu; 45.7%;  3. *TET2*: c.3955; 2A>C; p.?; 45.2% | 46,XX[20] |
| 78 | 78 | F | 3 | 1. *SF3B1*: c.1873C>T; p.Arg625Cys; 37.1%;  2. *TET2*: c.3967G>T; p.Glu1323*; 31.9% | 46,XX[20] |
| 79 | 86 | M | 3 | 1. *SF3B1*: c.1997A>C; p.Lys666Thr; 36.6% | 46,XY[20] |
| 80 | 73 | M | 3 | 1. *IDH2*: c.418C>G; p.Arg140Gly; 45.5.,1%;  2. *SRSF2*: c.284_307del; p.Pro95_Arg102del; 35% | 46,XY[20] |
| 81 | 71 | M | 3 | 1. *SF3B1*: c.2098A>G; p.Lys700Glu; 28.8%; | 46,XY[20] |
| 82 | 68 | M | 3 | 1. *TET2*: c.1763_1764insTA; p.Ile589Lysfs*13; 7.8%; 2. *SF3B1*: c.2098A>G; p.Lys700Glu; 33.9% | 46,XY[20] |
| 83 | 70 | M | 3 | 1. *SF3B1*: c.2098A>G; p.Lys700Glu; 38% | 46,XY[20] |
| 84 | 67 | M | 3 | 1. *DNMT3A*: c.2645G>A; p.Arg882His; 38.1%; 2. *SF3B1*: c.1986C>G; p.His662Gln; 37% | NA |
| 85 | 79 | M | 3 | 1. *TP53*: c.874A>T; p.Lys292*; 8% | 46,XY[20] |
| 86 | 69 | F | 3 | None | 46,XX[20] |
| 87 | 84 | M | 3 | 1. *TET2*: c.3500+1G>A; p.?; 41.3.1. *SRSF2*: c.284C>G; p.Pro95Arg; 42.1. | 46,XY[20] |
| 88 | 85 | M | 3 | 1. *RUNX1*: c.211del; p.Leu71Serfs*24; 43.1%;  2. *SRSF2*: c.284_307del; p.Pro95_Arg102del; 46.2%;  3. *TET2*: c.4639C>T; p.Gln1547*; 44%;  4. *TET2*: c.5062_5063del; p.Ser1688Phefs*4; 36.1% | 46,XY[20] |
| 89 | 81 | F | 3 | 1. *DNMT3A*: c.2536C>T; p.Gln846*; 44%;  2. *SF3B1*: c.2225G>A; p.Gly742Asp; 43%;  3. *TET2*: c.3575G>T; p.Gly1192Val; 39;1. | 46,XX[20] |
| 90 | 79 | M | 3 | 1. *SF3B1*: c.2098A>G; p.Lys700Glu; 9; | NA |
| 91 | 67 | M | 3 | 1. *DNMT3A*: c.2645G>A; p.Arg882His; 47.1%;  2. *SF3B1*: c.2098A>G; p.Lys700Glu; 9.3% | NA |
| 92 | 70 | M | 3 | 1. *DNMT3A*: c.2096G>A; p.Gly699Asp; 30.6%;  2. *IDH2*: c.419G>A; p.Arg140Gln; 6.8%;  3. *SF3B1*: c.2098A>G; p.Lys700Glu; 29.1% | 46,XY[20] |
| 93 | 54 | M | 3 | 1. *DNMT3A*: c.1192del; p.Ala398Profs*9; 45.1%; 2. *SF3B1*: c.2342A>G; p.Asp781Gly; 45.2% | 46,XY[20] |
| 94 | 64 | F | 3 | 1. *SF3B1*: c.2098A>G; p.Lys700Glu; 21.2%; 2. *TET2*: c.5079C>G; p.Tyr1693*; 23%; 3. *TET2*: c.1918C>T; p.Gln640*; 21.3% | 46,XX[20] |
| 95 | 75 | M | 3 | 1. *TET2*: c.3893G>A; p.Cys1298Tyr; 18.8%; 2. *SF3B1*: c.2098A>G; p.Lys700Glu; 26.8; | 46,XY[20] |
| 96 | 77 | F | 3 | 1. *ASXL1*: c.2367_2368insTT; p.Glu790Leufs*29; 8.6%;  2. *CBL*: c.1247G>A; p.Cys416Tyr; 6.1%;  3. *SF3B1*: c.2098A>G; p.Lys700Glu; 44.1% | 46,XX[20] |
| 97 | 67 | M | 4 | 1. *SF3B1*: c.1868A>G; p.Tyr623Cys; 47.7%;  2. *SRSF2*: c.284C>A; p.Pro95His; 45.2%;  3. *TET2*: c.812C>G; p.Ser271*; 27.7%;  4. *TET2*: c.4317dup; p.Arg1440Thrfs*38; 48.9% | 46,XY[20] |
| 98 | 59 | M | 4 | 1. *GATA2*: c.1166_1183del; p.Lys389_Gln394del; 23.1%;  2. *SF3B1*: c.2098A>G; p.Lys700Glu; 28% | 46,XY,+1,der1;7)q10;p10)[6]/47,idem,+8[14] |
| 99 | 77 | M | 4 | 1. *IDH1*: c.395G>A; p.Arg132His; 39.8%;  2. *SRSF2*: c.281_283dup; p.Arg94dup; 33.6%; | 46,XY[20] |
| 100 | 84 | M | 4 | 1. *SF3B1*: c.1996A>C; p.Lys666Gln; 29%;  2. *SRSF2*: c.283C>A; p.Pro95Thr; 48% | 47,XY,+20,del(20)(q11.2q13.3)x2[18]/46,XY[2] |
| 101 | 84 | M | 4 | 1. *TET2*: c.4133G>A; p.Cys1378Tyr; 38.1%;  2. *SF3B1*: c.1866G>T; p.Glu622Asp; 40% | 46,XY[20] |
| 102 | 74 | F | 4 | 1. *MPL*: c.1545G>A; p.Trp515*; 45%;  2. *SF3B1*: c.2098A>G; p.Lys700Glu; 16.1%;  3. *TET2*: c.3314_3315del; p.Ile1105Argfs*24; 17%;  4. *TET2*: c.5581G>A; p.Gly1861Arg; 18.1% | 46,XX[20] |
| 103 | 66 | M | 4 | 1. *DNMT3A*: c.2645G>A; p.Arg882His; 40.1%;  2. *SF3B1*: c.2098A>G; p.Lys700Glu; 41% | 47,XXY?c[20] |
| 104 | 76 | F | 4 | 1. *TET2*: c.4724del; p.Pro1575Glnfs*21; 9.6%;  2. *GATA2*: c.961C>T; p.Leu321Phe; 6.1%;  3. *SF3B1*: c.2098A>G; p.Lys700Glu; 10% | 46,XX,-2,-20,+2mar[2]/46,XX[18] |
| 105 | 65 | M | 5 | 1. *TP53*: c.749C>T; p.Pro250Leu; 23.8% | 46,XX,add(5)(q11.2)[14]/46,XX[6] |
| 106 | 87 | F | 5 | 1. *CBL*: c.1259G>A; p.Arg420Gln; 31.6% | 46,XX,add(5)(q11.2),del(11)(q23)[20] |
| 107 | 73 | M | 6 | None | 46,XY[20] |
| 108 | 84 | M | 6 | 1. *(RUNX1*: c.1034dup; p.Ser346Leufs*227; 39; | 46,XY[20] |
| 109 | 67 | M | 6 | 1. *TET2*: c.3410-2del; p.?; 28%; 2. *TET2*: c.3882C>G; p.Tyr1294*; 43.2%; 3. *ZRSR2*: c.376C>T; p.Arg126*; 15%; 4. *ZRSR2*: c.886_887del; p.Gln296Alafs*4; 22% | 46,XY[20] |
| 110 | 64 | F | 6 | 1. *BCOR*: c.4087_4088del; p.Cys1363Glnfs*45; 34.7%;  2. *GATA2*: c.700_703dup; p.Thr235Serfs*48; 31.2%;  3. *RUNX1*: c.567_568insCCCCC; p.Gly190Profs*22; 31.6%;  4. *U2AF1*: c.470A>G; p.Gln157Arg; 41.3% | 46,XX,der(7)t(1;7)(q21;q34)[4]/47,idem,+8[10]/46,XX[1] |
| 111 | 70 | M | 6 | 1. *ASXL1*: c.2250del; p.Val751Leufs*21; 39%;  2. *IDH2*: c.419G>A; p.Arg140Gln; 39.1%;  3. *SRSF2*: c.284C>A; p.Pro95His; 44% | 46,XY[20] |
| 112 | 78 | M | 6 | 1. *ASXL1*: c.1934dup; p.Gly646Trpfs*12; 41.1%;  2. *RUNX1*: c.416G>A; p.Arg139Gln; 43.1%;  3. *SRSF2*: c.284C>A; p.Pro95His; 42% | 46,XY[20] |
| 113 | 73 | M | 6 | 1. *ASXL1*: c.1934dup; p.Gly646Trpfs*12; 6.2% | 46,XY[20] |
| 114 | 75 | M | 6 | 1. *ASXL1*: c.1902_1924del; p.Glu635Argfs*15; 38.1%;  2. *RUNX1:* c.416G>A; p.Arg139Gln; 44.1%;  3. *SRSF2*: c.284C>A; p.Pro95His; 45% | 46,XY[20] |
| 115 | 70 | M | 6 | 1. *BCOR*: c.3958A>T; p.Lys1320*; 87%;  2. *DNMT3A*: c.2645G>A; p.Arg882His; 46;1%;  3. *U2AF1*: c.101C>T; p.Ser34Phe; 39% | 46,XY[20] |
| 116 | 75 | M | 6 | 1. *ASXL1*: c.1900_1922del;p.Glu635Argfs*15; 28%;  2. *EZH2*: c.908-1G>A; p.?; 78.3%;  3. *RUNX1*: c.397G>C; p.Asp133His; 35.5%;  4. *RUNX1*: c.253del; p.Leu85Cysfs*10; 39.2%;  5. *TET2*: c.3996del; p.Met1333Trpfs*30; 40.2%;  6. *ZRSR2*: c.208A>T; p.Arg70*; 84.7% | MDS FISH normal |
| 117 | 69 | M | 6 | 1. *ASXL1*: c.1934dup; p.Gly646Trpfs*12. 29.9%;  2. *GATA2*: c.1165_1173del; p.Lys389_Glu391del; 34.1%.  3. *ZRSR2*: c.883C>T; p.Arg295*; 77.4% | 46,XY[20] |
| 118 | 86 | M | 6 | 1. *ASXL1*: c.3131_3132del; p.Ser1044*; 39% 2. *SRSF2*: c.284C>A; p.Pro95His; 43.1%; 3. *SETBP1*: c.2602G>A; p.Asp868Asn; 42%. | 46,XY[20] |
| 119 | 72 | M | 6 | 1. *ASXL1*: c.2077C>T; p.Arg693*; 21. | 46,XY[20] |
| 120 | 80 | M | 6 | 1. *ASXL1*: c.2888dup; p. Ser964Ilefs*6; 11.5%;  2. *ASXL1*: c.2077C>T; p. Arg693*; 6%;  3. *CBL*: c.1247G>A; p. Cys416Tyr; 38.8%;  4. *ETV6*: c.306del; p. Arg103Alafs*19; 14.3%;  5. *ETV6*: c.1237_1238dup; p. Gln413Hisfs*9; 13.5%;  6. *JAK2*: c.1849G>T; p. Val617Phe ; 13.4%;  7. *U2AF1*: c.470A>C; p. Gln157Pro ; 37.7% | 44,X,-Y,-7[9]/45,X,-Y[11] |
| 121 | 77 | M | 6 | 1. *BCOR*: c.4144G>T; p.Glu1382*; 93%;  2. *DNMT3A*: c.2644C>T; p.Arg882Cys; 45.1%;  3. *KRAS*: c.38G>A; p.Gly13Asp; 44.1%;  4. *RUNX1*: c.416G>A; p.Arg139Gln; 47.1%;  5. *U2AF1*: c.101C>T; p.Ser34Phe; 43% | 47,XY,+8[20] |
| 122 | 69 | M | 6 | 1. *ASXL1*: c.1773C>G; p.Tyr591*; 24%;  2. *BCOR*: c.2726dup; p.Ala911Cysfs*6; 16%;  3. *BCOR*: c.3328_3329insT; p.Gln1110Leufs*32; 29%;  4. *NRAS*: c.35G>A; p.Gly12Asp; 7.1%;  5. *U2AF1*: c.101C>T; p.Ser34Phe; 21% | 46,XY,add(13)(p11.2)[4]/46,XY[14]. |
| 123 | 60 | M | 6 | 1. *ASXL1*: c.1934dup; p.Gly646Trpfs*12; 29%;  2. *BCOR*: c.4723dup; p.Met1575Asnfs*11; 38;2%;  3. *RUNX1*: c.683_686dup; p.Thr230Leufs*5; 25.1%;  4. *SRSF2*: c.284C>A; p.Pro95His; 39% | 46,XY[20] |
| 124 | 86 | M | 6 | 1. *ASXL1*: c.1934dup; p.Gly646Trpfs*12. 26.1%;  2. *RUNX1*: c.415C>T; p.Arg139*; 8.1%;  3. *SRSF2*: c.284C>A; p.Pro95His; 11% | 46,XY[20] |
| 125 | 73 | M | 6 | 1. *ASXL1*: c.1900_1922del; p.Glu635Argfs*15; 60%;  2. *EZH2*: c.118- 2A>G; p.?; 41.1%;  3. *NRAS*: c.35G>C; p.Gly12Ala; 43.1%;  4. *RUNX1*: c.541C>T; p.Gln181*; 45% | 46,XY[20] |
| 126 | 62 | M | 6 | 1. *ASXL1*: c.2483del; p. Gly828Alafs*10; 34%;  2. *IDH2*: c.419G>A; p. Arg140Gln; 48.5%;  3. *JAK2*: c.1849G>T; p. Val617Phe; 17.1%;  4. *SRSF2*: c.284C>G; p. Pro95Arg; 49% | 46,XY,t(7;13)(p15;q14)[4]/46,XY[16] |
| 127 | 78 | M | 6 | None | 46,XY[20] |
| 128 | 71 | M | 6 | 1. *TP53*: c.481G>A; p.Ala161Thr; 42%. | 43-47,X,-Y,add(3)(p11),del(5)(q13q33),-7,+8,psu dic(12;20)(p11.2;q11.2),add(16)(q13),-18,-20, +add(20)(q13.3),+0-2mar[cp20] |
| 129 | 79 | M | 6 | 1. *GATA2*: c.750_755delinsT; p.Ser251Cysfs*29; 37.6%;  2. *SF3B1*: c.2098A>G; p.Lys700Glu; 46% | 46,Y,t(X;8)(q22;q24.1)[12]/46,XY[8] |
| 130 | 86 | M | 6 | 1. *DNMT3A*: c.1084C>T; p.Gln362*; 43.2%;  2. *TP53*: c.743G>A; p.Arg248Gln; 42%;  3. *TP53*: c.637C>T; p.Arg213*; 41% | 44-49,XY,del(5)(q22q35),-7,dic(12;22)(p11.2;p12),-18,+add(20)(q13.1),+0-5r[cp9]/44-47,sl,-12,-dic(12;22),-add(20),der(20)t(18;20)(q11.2;q13.1)[8]/44-45,sl,-dic(12;22),dic(12;22)(p13;p11.2)dup(12)(p13p11.2)[2]/45,XY,del(5)(q22q35),-7,-12,der (22)t(12;22)(q15;p11.2),+3r[1] |
| 131 | 76 | M | 6 | 1. *RUNX1*: c.521G>A; p.Arg174Gln; 37.1%;  2. *SF3B1*: c.1998G>T; p.Lys666Asn; 42%;  3. *TET2*: c.3058C>T; p.Gln1020*; 41.1% | 46,XY[20] |
| 132 | 68 | M | 6 | 1. *DNMT3A*: c.2645G>A; p.Arg882His; 14.2%;  2. *PHF6*: c.941T>C; p.Ile314Thr; 20.6%;  3. *TET2*: c.1636A>T; p.Lys546*; 11.4%;  4. *TET2*: c.3921del; p.Lys1308Serfs*55; 13.1% | 46,XY[20] |
| 133 | 77 | M | 6 | 1. *BCOR*: c.4957del; p.Gln1653Lysfs*21; 42.9%; 2. *BCOR*: c.4981C>T; p.Arg1661*; 5.6%; 3. *SF3B1*: c.1873C>T; p.Arg625Cys; 29.5% | 46,XY[20] |
| 134 | 85 | M | 6 | 1. *TP53:* c.584T>C; p.Ile195Thr; 35% | 42-47,XY,-5,add(6)(p21.3),der(7;18)(p10;q10),der(9)t(9;13)(p 13;q14), -13,-18,add(20)(q11.2),-22,+1-3r, +2-4mar[cp6]/46,XY[14] |
| 135 | 65 | M | 6 | 1. *BCOR*: c.1024C>T; p.Arg342*; 24%;  2. *DNMT3A*: c.2645G>A; p.Arg882His; 10%;  3. *PTPN11*: c.214G>A; p.Ala72Thr; 10%;  4. *RUNX1*: c.1035_1038dup; p.Ala347Leufs*227; .9.1%;  5. *U2AF1*: c.101C>T; p.Ser34Phe; 11% | 46,XY,t(3;19)(p21;q13.1)[6]/46,XY[14] |
| 136 | 65 | F | 6 | 1. *SF3B1:* c.2098A>G; p.Lys700Glu; 40.8% | 46,XX[20] |
| 137 | 61 | F | 6 | 1. *DNMT3A*: c.1015; 24_1015del; p.?; 26%; 2. *NPM1*: c.860_863dup; p.Trp288Cysfs*12; 16% | 47,XX,+8[18]/46,XX[2]. |
| 138 | 80 | M | 7 | 1.*DNMT3A*: c.2384G>C; p.Trp795Ser; 25.2. 2. *SF3B1*: c.1984C>T; p.His662Tyr; 29.9%; 3. *SETBP1*: c.2602G>T; p.Asp868Tyr; 30.5%; 4. *TP53*: c.707A>G; p.Tyr236Cys; 41.1%; 5. *U2AF1*: c.470A>G; p.Gln157Arg; 28.1% | 46-47,XY,+add(3)(q12),der(5)t3;5)q21;q13),-17,add20)q13.3),+0-1r [cp6]/46,XY[14] |
| 139 | 59 | F | 7 | 1.*RUNX1*: c.1283_1286dup; p.Glu429Aspfs*145; 38.6% | 45,X,-X,t(3;5)(q25;q35)[18]/46,XX[2] |
| 140 | 80 | M | 7 | 1. *ASXL1*: c.2324T>G; p.Leu775*; 17%; 2. *TET2*: c.961C>T; p.Gln321*; 25.3%; 3. *U2AF1*: c.467G>A; p.Arg156His; 9.6% | 47,XY,+21[3]/46,XY[17] |
| 141 | 66 | M | 7 | 1. *TP53*: c.586C>T; p.Arg196*; 84.9% | 44,X,-Y,add(1)(q42),add(5)(q13),add(7)(q22),inv(11)(p15q11), add(11)(p15),add(12)(p13),-13,- 22,+mar[19]/46,XY[1] |
| 142 | 85 | M | 7 | 1. *ASXL1*: c.1934dup; p.Gly646Trpfs*12. 31.3%; 2. *IDH2*: c.419G>A; p.Arg140Gln; 39.7%; 3.*RUNX1*: c.887; 2A>G; p.?; 5.6%; | 46,XY[20] |
| 143 | 78 | F | 7 | 1. *RUNX1:* c.232C>A; p.His78Asn; 39.3%; 2. *CSF3R*: c.2255dup; p.Tyr752*;18.9% | 45,XX,-7[3]/46,XX,-7,+r[16]/46,XX[1] |
| 144 | 61 | M | 7 | None | 46-48,XY,add(3)(q27),del(5)(q13q33),+9,der(9)t(9;15)(q21;q22)x2,inv(10)(p13q26),-15,add(20) (p11.2),+22,+0-1mar[cp17]/46,XY[3] |
| 145 | 60 | M | 7 | 1. *TP53*: c.658T>C; p.Tyr220His; 87; | 44-45,XY,del(4)(q21q31),-5,-7,idic(12;14)(p11.2;p11.2),+14,+0-1r[cp20] |
| 146 | 69 | M | 7 | 1. *TP53*: c.578A>C; p.His193Pro; 20; | 43,XY,-2,t(2;17)(p11.2;p11.2),add(3)(p21),add(5)(q13),der(7;12)(p10; q10),del(8)(q21q24),- 10,der(11)t(10;11)(q11.2;q21),-13,-16,+2mar[15]/46,XY[5] |
| 147 | 59 | F | 7 | 1. *IDH2*: c.419G>A; p.Arg140Gln; 9.2%; 2. *RUNX1*: c.1283_1286dup; p.Glu429Aspfs*145; 37.8% | 45,X,-X,t(3;5)(q25;q35)[15]/46,XX[5] |
| 148 | 76 | F | 7 | 1. *TP53*: c.832C>G; p.Pro278Ala; 5.1%; 2. *SF3B1*: c.2098A>G; p.Lys700Glu; 20% | 46,XX,del(5)(q31q35)[1]/46,idem,-7,del(20)(q11.2q13.3)[5]/46,XX,del(  5)(q13q33)[3]/47,XX,  +8[3]/46,XX[8] |
| 149 | 59 | F | 7 | 1. *RUNX1*: c.1283_1286dup; p.Glu429Aspfs*145; 16% | 45,X,-X,t(3;5)(q25;q35)[4]\46,XX[16] |
| 150 | 72 | F | 7 | 1. *NRAS*: c.35G>A; p.Gly12Asp; 28%; 2. *NRAS*: c.35G>C; p.Gly12Ala; 13.1%; 3. *SRSF2*: c.284C>T; p.Pro95Leu; 47.2%; 4. *TET2*: c.3804-2A>T; p.?; 47%; 5. *TET2*: c.3861del; p.Phe1287Leufs*76; 45% | 47,XX,+8[12]/46,XX[8] |
| 151 | 68 | F | 7 | 1. *NPM1*: c.863_864insCATG; p.Trp288Cysfs*12; 37% | 46,XX[20] |
| 152 | 90 | F | 7 | 1. *BCOR*: c.4639C>T; p.Arg1547*; 21.3%; 2. *PHF6*: c.57_58dup; p.Cys20Phefs*14. 5.2%; 3. *RUNX1*: c.403A>G; p.Arg135Gly; 22.4%; 4. *SRSF2*: c.284C>T; p.Pro95Leu; 25.9% | 47,XX,+8[2]/46,XX[18] |
| 153 | 70 | M | 7 | None | 46-50,XY,+1,del(4)(q21q25),+6,+8,-10,add(12)(q24.3),add(19)(p13.1),add(21)(q22),+1-2mar[cp9]/46,XY[11] |
| 154 | 68 | F | 7 | 1. *ASXL1*: c.1773_1778del; p.Tyr591*; 20.1%; 2. *CSF3R*: c.2245C>T; p.Gln749*; 25% | 46,XX[20] |
| 155 | 77 | M | 7 | 1. *U2AF1*: c.470A>C; p.Gln157Pro; 36.5% | 46,XY,del(7)(q22),inv(12)(p13q13),del(13)(q12q14)[1]/46,idem,t(11;19)(q23;p13.1)[cp19] |
| 156 | 85 | F | 7 | 1. *ASXL1*: c.1900_1922del; p.Glu635Argfs*15; 11.1%;  2. *NRAS*: c.37G>C;p.Gly13Arg; 13.4%;  3. *RUNX1*: c.529C>T;p.Arg177*; 26.4%;  4. *SRSF2*: c.284C>A;p.Pro95His; 29.5% | 46,XX,add(18)(p11.2)[20] |
| 157 | 64 | F | 7 | 1. *CBL*: c.1151G>A; p.Cys384Tyr; 52% | 46,XX,+1,add(1)(p13)x2,-11,-13,der(22)t(13;22)(q12;q11.2),+mar[20] |
| 158 | 71 | M | 7 | 1. *TP53*: c.659A>G; p.Tyr220Cys; 20% | 43-45,Y,-X,add(3)(q21),add(4)q31.1),-5,-6,-7,-14,add(17)(p1 1.2),add(20)(q13.1),add(21)(p11.2),+2-4mar[cp15]/67,XY,+1,+2 ,+4,-5,+add(6)(q21),add(7)(q32),+8,+9,+10,+10,+11,+13,+14,+15,+16,+18,+19,+20,+20,+add(21)(p11.2),+22,+22,+2-3mar[cp5] |
| 159 | 75 | M | 7 | None | 46,XY[20] |
| 160 | 61 | M | 7 | 1. *DNMT3A*: c.2645G>A; p.Arg882His; 10%; 2. *IDH1*: c.394C>T; p.Arg132Cys; 5% | 46,XY[20] |
| 161 | 56 | F | 7 | 1. *TP53*: c.800G>C; p.Arg267Pro; 45% | 45-46,XX,add(3)(q11.2),add(5)(q11.2),dic(6;17)(p21.3;p11.2),+9,der(9)t(9;13)(q22;q14),-13,-16,add(16)(p13.3),dic(16;21)(p1.3;q22),add(17)(p11.2),add(21)(q22),del(21)(q22),psu dic(21;16)(q22;p13.3),i(22)(q10),+0-1r[cp19]/46,XX[1] |
| 162 | 69 | M | 7 | 1. *PHF6*: c.27dup; p.Gly10Argfs*12; 6% | 46,XY[20] |
| 163 | 79 | M | 7 | 1. *ASXL1*: c.1281dup; p.Gln428Thrfs*10; 36.2%; 2. *GATA2*: c.170del; p.Asn57Thrfs*23; 36%; 3. *GATA2*: c.1168_1170del; p.Lys390del; 33.2%. 4. *SRSF2*: c.284C>T; p.Pro95Leu; 32%; 5. *U2AF1*: c.470A>G; p.Gln157Arg; 24. | 45,XY,-7[6]/46,XY,del(7)(q11.2)[1]/46,XY[13] |
| 164 | 75 | F | 7 | 1. *ASXL1*: c.2077C>T; p.Arg693*; 19% | 46,XX[20] |
| 165 | 80 | M | 7 | 1. *ASXL1*: c.1934dup; p.Gly646Trpfs*12; 42%; 2. *CEBPA*: c.700_710dup; p.Pro239Argfs*83; 23.1%; 3. *NRAS*: c.35G>A; p.Gly12Asp; 14.1%; 4. *SRSF2*: c.284C>T; p.Pro95Leu; 46%; 5.*TET2*: c.2113C>T; p.Gln705*; 46.1% | 46,XY,del(20)(q11.2q13.3)[20] |
| 166 | 70 | M | 7 | None | 46,XY[20] |
| 167 | 82 | F | 7 | 1. *ASXL1*: c.1934dup; p.Gly646Trpfs*12; 36.4%; 2. *EZH2*: c.545dup; p.Asn182Lysfs*; 15.7%; 3. *IDH2*: c.419G>A; p.Arg140Gln; 37.4%; 4. *U2AF1*: c.470A>C; p.Gln157Pro; 24.2% | 46,XX[20] |
| 168 | 85 | M | 7 | 1. *ASXL1*: c.1934dup; p.Gly646Trpfs*12; 35%; 2. *TET2*: c.3646C>T; p.Arg1216*; 42%; 3. *TET2*: c.3803+5G>C ; 44.1%; 4. *SRSF2*: c.284C>A; p.Pro95His; 43% | 47,XY,+8[5]/46,XY[15] |
| 169 | 67 | F | 7 | 1. *TP53*: c.625del; p.Arg209Glufs*38; 31% | 43-46,XX,-4,del(5)(q14q33),-17,add(21)(q22),-22,+1~4mar[cp10]/44,idem,add(14)(p10),-18cp3]/43-45,idem,add(5)(p13),-18[cp2]/44-46,idem,del(7)(q22q34)[cp4]/43-45,idem,add(7)(q21),-15,der(15)t(15;15)(p11.1;q15,-18[cp5] |
| 170 | 50 | M | 7 | 1. *ASXL1*: c.2077C>T; p.Arg693*; 50%; 2. *JAK2*: c.1849G>T; p.Val617Phe; 18% | 46,XY[20] |
| 171 | 74 | M | 7 | 1. *BCOR*: c.1328T>A; p.Leu443*; 75%; 2. *BCOR*: c.893del; p.Pro298Leufs*80; 5%; 3. *DNMT3A*: c.2560G>T; p.Glu854*; 86%; 4. *IDH2*: c.419G>A; p.Arg140Gln; 42.1%; 5. *CEBPA*: c.543_545del; p.Tyr181*; 43.1%; 6. *PHF6*: c.636T>A; p.Cys212*; 83% | 47, XY,+8[3]/46,XY[17] |
| 172 | 75 | M | 8 | 1. *ASXL1*: c.1534C>T; p.Gln512*; 39%; 2. *CBL*: c.1111T>C; p.Tyr371His; 43. 3. *EZH2*: c.1212_1216del; p.Lys405Argfs*2. 35%; 4. *EZH2*: c.2110+1G>A; p.? 35.1% | 45,X,-Y[4]/45,idem,del(7)(p11.2)[16] |
| 173 | 83 | M | 8 | 1. *ASXL1*: c.2077C>T; p.Arg693*; 46.1%; 2. *CBL:* c.1096-1G>T; p.?; 20.1%; 3. *SETBP1*: c.2602G>A; p.Asp868Asn; 46%; 4. *SRSF2*: c.284C>A; p.Pro95His; 47.1% | 46,XY,add(12)(p11.2)[5]/46,XY[15] |
| 174 | 77 | M | 8 | 1. *BCOR*: c.4957del; p.Gln1653Lysfs*21; 41%; 2. *BCOR*: c.4981C>T; p.Arg1661*; 5.1%; 3. *SF3B1*: c.1873C>T; p.Arg625Cys; 38% | 46,XY[20] |
| 175 | 71 | M | 8 | 1. *U2AF1*: c.101C>T; p.Ser34Phe; 41% | 46,XY,del(20)(q11.2q13.3)[1]/46,XY[19]. |
| 176 | 82 | M | 8 | 1. *PHF6*: c.88C>T; p.Gln30*; 57%; 2. *PHF6*: c.407del; p.His136Leufs*7; 7.2%; 3. *TET2*: c.4249G>T; p.Val1417Phe; 44%; 4. *TET2*: c.4546C>T; p.Arg1516*; 40.1%; 5. *RUNX1*: c.38_92del; p.Phe13Trpfs*14. 7.5% | 45,X,-Y[20] |
| 177 | 77 | M | 9 | 1. *TP53*: c.722C>T; p.Ser241Phe; 52.3% | 55,XY,+1,+4,+6,+8,+9,+10,+11,+19,+21[5]/55,sl,+add(4)(q21),-5[4]/54,  sl,der(8)t(8;13)  (p21;q14),del(11)(p11.2),-13[4]/55,sl,del(13)(q12q14)[2]/46,XY[5] |
| 178 | 76 | M | 9 | 1. *U2AF1*: c.101C>T; p.Ser34Phe; 29% | 46,XY,del(20)(q11.2q13.3)[18]/46,XY[2] |
| 179 | 70 | F | 9 | 1. *TP53*: c.473G>A; p.Arg158His; 46%; 2. *TP53*: c.824G>A; p.Cys275Tyr; 45% | 46,XX,add(3)(p13),-5,+8[7]/46,idem,add(12)(p11.2),add(12)(q24.1),add(18)(p11.2)[13] |
| 180 | 59 | F | 9 | 1. *TP53*: c.716_717insT; p.Ser240Glnfs*24; 39%; | 44,XX,der(5;18)(p10;q10),der(14;17)(q10;q10)[16]/45,idem,+8[ 2]/46,XX[2] |
| 181 | 72 | M | 9 | None | 46,XY,inv(13)(q12q32)[2]/47,XY,+8[2]/46,XY[16] |
| 182 | 67 | M | 9 | 1. *TP53*: c.818G>A; p.Arg273His; 93%. | 44-47,XY,+5,dic(5;8)(q11.2;p21)x2,-6,der(6)add(6)(p21.1)t(6;13)(q23;q12),+8,del(8)(p11.2),-13,+1-3mar[cp20] |
| 183 | 72 | M | 9 | 1. *TP53*: c.527G>A; p. Cys176Tyr; 26.7%; 2. *TP53*: c.743G>A; p. Arg248Gln; 24.4% | 44,XY,add(5)(q11.2),-7,-12,der(18)t(7;18)(p15;q11.2)[15]/45,XY,-7,ad d(14)(q22),der(18) t(7;18)(p15;q11.2),add(21)(q22)[5] |
| 184 | 72 | M | 9 | 1. *RUNX1*: c.466_467dup; p.Pro157Hisfs*28; 33.1%; 2. *U2AF1*: c.470A>G; p.Gln157Arg; 33% | 46,XY,+1,der(1;7)(q10;p10)[14]/47,sl,+8[1]/49,sl,+19,+21,+21 [1]/46,XY[4] |
| 185 | 64 | M | 9 | 1. *DNMT3A*: c.1198G>T; p.Glu400*; 29.1% | 46,XY[20] |
| 186 | 20 | M | 9 | None | 45,XY,-7[12]/46,XY[8] |
| 187 | 77 | F | 9 | 1. *ASXL1*: c.2177del; p.Lys726Argfs*18; 27.2%; 2. *TET2*: c.3895A>T; p.Lys1299*; 25.5%; 3. *U2AF1*: c.472_477dup; p.Tyr158_Glu159dup; 22.8% | NA |
| 188 | 68 | M | 9 | 1. *KRAS*: c.64C>A; p.Gln22Lys; 39.4% | 46,XY,−2,t(3;5)(q21;q22),der(4)t(2;4)(p11.2;q31.3),−20,+der(?)t(?;2)(?;q11.2),+mar[cp8] |
| 189 | 68 | M | 2 | 1. *ASXL1*: c.1934dup; p.Gly646Trpfs*12; 36.1%;  2. *IDH1*: c.395G>A; p.Arg132His; 44.2%;  3. *KRAS*: c.182A>G; p.Gln61Arg; 40.8%;  4. *SRSF2*: c.284C>A; p.Pro95His; 43.6% | 46, XY[20] |
| 190 | 50 | M | 8 | None | 46,XY,add(2)(p11.2),add(5)(q31),-17,+mar[3]/46,XY[17] |
| 191 | 57 | F | 10 | None | 46,XX[20] |
| 192 | 83 | M | 10 | 1. *ZRSR2*: c.1049_1055dup; p.Thr353Argfs*34; 62.2% | 46,XY[20] |
| 193 | 73 | M | 10 | 1. *SF3B1*: c.2098A>G; p.Lys700Glu; 26.2% | 46,XY[20] |
| 194 | 85 | M | 10 | 1. *DNMT3A*: c.1143_1158dup; p.Cys387Glufs*11; 23.8%; 2. *TET2*: c.687_688del; p.Leu230Valfs*23; 34.2%; 3. *TET2*: c.1692G>A; p.Trp564*; 44% | 46,XY[20] |
| 195 | 73 | F | 10 | None | 46,XX[20] |
| 196 | 58 | F | 10 | None | 46,XX[20] |
| 197 | 56 | M | 10 | None | 46,XY[20] |
| 198 | 86 | M | 10 | None | 46,XY[20] |
| 199 | 84 | M | 10 | 1. *ZRSR2*: c.827+2dup;87% | 46,XY[20] |
| 200 | 57 | F | 10 | None | 46,XX[20] |
| 201 | 45 | F | 10 | None | 46,XX[20] |
| 202 | 57 | M | 10 | 1. *DNMT3A*: c.1668-2A>G; p.?; 17% | 46,XY,del(20)(q11.2q13.1)[4]/46,XY[16] |
| 203 | 50 | F | 10 | None | 46,XX[20] |
| 204 | 69 | M | 10 | 1. *TET2*: c.651del; p.Val218Trpfs*32; 8%; 2. *TET2*: c.2065del; p.Ser689Profs*11; 54%; 3. *TET2*: c.4661_4664del; p.Thr1554Serfs*16; 14% | 46,XY[20] |
| 205 | 55 | F | 10 | None | 46,XX[20] |
| 206 | 87 | M | 10 | None | 45,X,-Y[4]/46,XY[16] |
| 207 | 77 | F | 10 | None | 46,XX[20] |
| 208 | 67 | F | 10 | 1. *IDH1*: c.394C>A; p.Arg132Ser; 29.3%; 2. *SRSF2*: c.284C>T; p.Pro95Leu; 36% | 46,XX[20] |
| 209 | 51 | F | 10 | None | 46,XX[20] |
| 210 | 46 | F | 10 | None | 46,XX[20] |
| 211 | 78 | F | 10 | None | 46,XX[20] |
| 212 | 72 | M | 10 | 1. *SF3B1*: c.2342A>G; p.Asp781Gly;26.7; | 46,XY[20] |
| 213 | 63 | M | 10 | None | 46,XY,del(20)(q11.2q13.3)[1]/46,XY[19] |
| 214 | 50 | M | 10 | None | 46,XY[20] |
| 215 | 42 | F | 10 | None | 46,XX[20]. |
| 216 | 83 | M | 10 | 1. *ASXL1*: c.1758dup; p.Gly587Argfs*32; 42.1%; 2. *U2AF1*: c.470A>C; p.Gln157Pro; 42.3% | 46,XY[20] |
| 217 | 76 | M | 10 | 1. *SF3B1*: c.2098A>G; p.Lys700Glu; 7.6% | 46,XY[20] |
| 218 | 65.0 | M | 10 | 1. *DNMT3A*: c.1903C>T; p.Arg635Trp; 12.4% | 46,XY[10] |
| 219 | 24 | M | 10 | None | 46,XY[20] |
| 220 | 74 | F | 10 | 1. *SF3B1*: c.2098A>G; p.Lys700Glu; 6.3% | del13q[6] |
| 221 | 64 | M | 10 | None | 46,XY[20] |
| 222 | 61 | M | 10 | None | 46,XY[20] |
| 223 | 84 | M | 10 | None | 45,X,-Y[4]/46,XY[16] |
| 224 | 63 | F | 10 | None | 46,XX[20] |
| 225 | 73 | M | 10 | 1. *U2AF1*: c.101C>T; p.Ser34Phe; 22.4% | 46,XY[20] |
| 226 | 64 | M | 10 | None | 46,XY[20] |
| 227 | 60 | M | 10 | None | 46,XY[20] |
| 228 | 54 | M | 10 | None | 46,XY[20] |
| 229 | 48 | F | 10 | None | 46,XX[20] |
| 230 | 71 | M | 10 | None | 46,XY[20] |
| 231 | 86 | M | 10 | None | 46,XY[20] |
| 232 | 66 | M | 10 | None | 46,XY[20] |
| 233 | 34 | M | 10 | None | 46,XY[20] |
| 234 | 64 | M | 10 | None | 46,XY[20] |
| 235 | 46 | F | 10 | None | 46,XX[20] |
| 236 | 69 | F | 10 | 1. *ASXL1*: c.1900_1922del; p.Glu635Argfs*15; 21% | 46,XX[20] |
| 237 | 72 | M | 10 | 1. *ZRSR2*: c.1122C>G; p.Tyr374*; 73% | 46,XY[20] |
| 238 | 54 | F | 10 | None | 46,XX[20] |
| 239 | 44 | F | 10 | None | 46,XX[20] |
| 240 | 79 | M | 10 | None | 45,X,-Y[9]/46,XY[11] |
| 241 | 41 | M | 10 | None | 46,XY[20] |
| 242 | 58 | F | 10 | 1. *TET2*: c.1771del; p.Gln591Serfs*10; 6.2% | 46,XX[20] |
| 243 | 75 | M | 10 | None | 46,XY[20] |
| 244 | 43 | M | 10 | None | 46,XY[20] |
| 245 | 77 | F | 10 | None | 46,XX[20] |
| 246 | 62 | M | 10 | None | 46,XY[20] |
| 247 | 76 | M | 10 | None | 46,XY[20] |
| 248 | 71 | F | 10 | None | 46,XX[20] |
| 249 | 65 | M | 10 | None | 46,XY[20] |
| 250 | 56 | F | 10 | None | 46,XX[20] |
| 251 | 71 | F | 10 | 1. *ASXL1*: c.2423del; p.Pro808Leufs*10; 11.9% | 46,XX[20] |
| 252 | 35 | M | 10 | None | 46,XY[20] |
| 253 | 38 | M | 10 | None | 47,XY,+8[20] |
| 254 | 46 | M | 10 | None | 46,XY[20] |
| 255 | 72 | M | 10 | None | 46,XY[20] |
| 256 | 72 | F | 10 | None | 45,X,-X[13]/46,XX[7] |
| 257 | 71 | F | 10 | None | 46,XX[20] |
| 258 | 72 | F | 10 | None | 46,XX[20] |
| 259 | 62 | F | 10 | None | 46,XX[20] |
| 260 | 80 | M | 10 | None | 45,X,-Y[7]/46,XY[13] |
| 261 | 62 | M | 10 | None | 46,XY[20] |
| 262 | 83 | F | 10 | 1. *TET2*: c.1207C>T; p.Gln403*; 43% | 46,XX[20] |
| 263 | 74 | F | 10 | None | 46,XX[20] |
| 264 | 60 | F | 10 | None | 46,XX[20] |
| 265 | 21 | M | 10 | None | 46,XY[20] |
| 266 | 78 | M | 10 | 1. *TET2*: c.421_424dup; p. Ser142Cysfs* ;17.1%; 2. *TET2*: c.3594+2dup; p.?; 23.7%; 3. *ZRSR2*: c.376C>T; p. Arg126*; 56.6% | 46,XY[20] |
| 267 | 52 | F | 10 | None | 46,XX,del(10)(q11.2q22)[20] |
| 268 | 14 | F | 10 | None | 46,XX[20] |
| 269 | 75 | M | 10 | 1. *SRSF2*: c.284_307del; p.Pro95_Arg102del; 48.2%; 2. *TET2*: c.543del; p.Leu182*;13%; 3. *TET2*: c.4080_4083del; p.Gly1361*; 40% | 46,XY[20] |
| 270 | 44 | M | 10 | None | 46,XY[20] |
| 271 | 46 | M | 10 | None | MDS FISH neg |
| 272 | 40 | F | 10 | None | 46,XX[20] |
| 273 | 66 | M | 10 | None | 46,XY[20] |
| 274 | 59 | F | 10 | None | 46,XX[20] |
| 275 | 80 | M | 10 | 1. *ASXL1*: c.1934dup; p.Gly646Trpfs*12; 12% | 46,XY,del(1)(p13p31.2)[17]/46,XY[3] |
| 276 | 76 | F | 10 | None | 46,XX[2] |
| 277 | 77 | F | 10 | 1. *TET2*: c.2539C>T; p.Gln847*; 41% | 46,XX[20] |
| 278 | 21 | M | 10 | None | 46,XY[20] |
| 279 | 52 | M | 10 | None | 46,XY[20] |
| 280 | 42 | M | 10 | None | 46,XY[20] |
| 281 | 82 | F | 10 | None | 46,XX[20] |
| 282 | 36 | F | 10 | None | 46,XX[20] |
| 283 | 85 | M | 10 | None | 45,X,-Y[3]/46,XY[17] |
| 284 | 66 | F | 10 | None | 46,XX[20] |
| 285 | 66 | M | 10 | 1. *TP53*: c.742C>T; p.Arg248Trp; 8% | 46,XY[20] |
| 286 | 58 | M | 10 | None | 46,XY[20] |
| 287 | 83 | M | 10 | None | 46,XY[20] |
| 288 | 46 | F | 10 | None | 46,XX[20] |
| 289 | 71 | M | 10 | None | 46,XY[20] |
| 290 | 62 | F | 10 | None | 46,XX[20] |
| 291 | 71 | M | 10 | None | 46,XY[20] |
| 292 | 82 | F | 10 | None | 46,XX[20] |
| 293 | 76 | M | 10 | None | 46,XY[20] |
| 294 | 27 | M | 10 | None | 46,XY[20] |
| 295 | 57 | F | 10 | None | 46,XX[20] |
| 296 | 66 | M | 10 | None | 46,XY[20] |
| 297 | 71 | M | 10 | None | 46,XY[20] |
| 298 | 38 | M | 10 | None | 46,XY[20] |
| 299 | 70 | F | 10 | None | 46,XX[20] |
| 300 | 57 | M | 10 | None | 46,XY[10] |
| 301 | 50 | F | 10 | None | 46,XX[20] |
| 302 | 57 | F | 10 | None | 46,XX[20] |
| 303 | 42 | M | 10 | None | 46,XY[20] |
| 304 | 77 | M | 10 | 1. *TET2*: c.4523_4524del; p.Ala1508Glufs*69; 22%; 2. *ZRSR2*: c.787G>T; p.Glu263*; 52% | 46,XY[20] |
| 305 | 66 | M | 10 | 1. *DNMT3A*: c.1555-3T>G; p.?; 17.2% | 46,XY[20] |
| 306 | 19 | M | 10 | 1. *ASXL1*: c.2638up; p.Thr880Asnfs*2; 11% | 46,XY[20] |

Disease category code: 1. MDS with single lineage dysplasia (MDS-SLD); 2. , MDS with multilineage dysplasia (MDS-MLD); 3. MDS with ring sideroblasts with single lineage dysplasia (MDS-RS-SLD); 4. MDS with ring sideroblasts with multilineage dysplasia (MDS-RS-MLD); 5. MDS with isolated del(5q); 6. MDS with excess blasts-1 (MDS-EB1); 7. MDS with excess blasts-2 (MDS-EB2); 8. MDS, unclassifiable (MDS-U); 9. therapy-related MDS (MDS-T); 10. Cytopenia cases not meeting the diagnostic criteria of MDS or other myeloid neoplasms (noMN).

Supplementary table 2. Clinical and genetic features and MN progression in noMN patients harboring mutations (CCUS) and/or cytogenetic abnormalities.

| **Case #** | **Age** | **Gender** | **Cytopenia Category** | **Cytogenetic results** | **Mutation summary** | **Progressed to MN** | **MN diagnosis** | **Time to MN progression (Months)** | **MN cytogenetic results** | **MN mutation summary** | **Time to final follow-up (Months)** |
| --- | --- | --- | --- | --- | --- | --- | --- | --- | --- | --- | --- |
| 192^§^ | 83 | M | 1 | 46,XY[20] | 1. *ZRSR2*: c.1049_1055dup; p.Thr353Argfs*34; 62.2% | Yes | Low-grade MDS | 10.3 | ND | ND | 60.8 |
| 216^§◊^ | 83 | M | 1, 3 | 46,XY[20] | 1. *ASXL1*: c.1758dup; p.Gly587Argfs*32; 42.1%; 2. *U2AF1*: c.470A>C; p.Gln157Pro; 42.3% | Yes | MDS-MLD | 49.7 | 46,XY[20] | 1. *ASXL1*:c.1758dup; p.Gly587Argfs*32 44%; 2. *U2AF*1:c.470A>C; p.Gln157Pro, 44% | 62 |
| 204^§^ | 69 | M | 4 | 46,XY[20] | 1. *TET2*: c.651del; p.Val218Trpfs*32; 8%; 2. *TET2*: c.2065del; p.Ser689Profs*11; 54%; 3. *TET2*: c.4661_4664del; p.Thr1554Serfs*16; 14% | Yes | MDS-U | 35.2 | 46,XY[20] | 1. *TET2*: c.651del; p.Val218Trpfs*32; 6%; 2. *TET2:* c.2065del; p.Ser689Profs*11; 63%; 3. *TET2*:c.4661_ 4664del; p.Thr1554 Serfs*16; 11% | 50.8 |
| 208^§^ | 67 | F | 1, 2 | 46,XX[20] | 1. *IDH1*: c.394C>A; p.Arg132Ser; 29.3%; 2. *SRSF2*: c.284C>T; p.Pro95Leu; 36% | Yes | CMML-1 | 25.2 | ND | ND | 32.9 |
| 193^§^ | 73 | M | 4 | 46,XY[20] | 1. *SF3B1*: c.2098A>G; p.Lys700Glu;26.2% | Yes | MDS-MLD | 6.1 | 46, XY[20] | ND | 13.2 |
| 225^§^ | 73 | M | 4 | 46,XY[20] | 1. *U2AF1*: c.101C>T; p.Ser34Phe; 22.4% | Yes | MDS-U | 32.4 | 46,XY,del(13)(q12q22)[4]/45,X,-Y[6]/46,XY  [10] | ND | 59.8 |
| 237^§◊^ | 72 | M | 1 | 46,XY[20] | 1. *ZRSR2*: c.1122C>G; p.Tyr374*; 73% | Yes | MDS-SLD | 15.1 | karyotype ND; MDS FISH normal | 1. *ZRSR2*: c.1122C>G; p.Tyr374*; 81% | 61 |
| 266^§◊^ | 78 | M | 4 | 46,XY[20] | 1. *TET2*: c.421_424dup; p. Ser142Cysfs* ;17.1%; 2. *TET2*: c.3594+2dup; p.?; 23.7%; 3. *ZRSR2*: c.376C>T; p. Arg126*; 56.6% | Yes | MDS-MLD | 4.2 | 46,XY[20] | ND | 31.1 |
| 269^§^ | 75 | M | 1, 3 | 46,XY[20] | 1. *SRSF2*: c.284_307del; p.Pro95_Arg102del; 48.2%; 2. *TET2*: c.543del; p.Leu182*;13%; 3. *TET2*: c.4080_4083del; p.Gly1361*; 40% | Yes | MDS-MLD | 19.5 | 46, XY[20] | ND | 56.5 |
| 304^§^ | 77 | M | 4 | 46,XY[20] | 1. *TET2*: c.4523_4524del; p.Ala1508Glufs*69; 22%; 2. *ZRSR2*: c.787G>T; p.Glu263*; 52% | Yes | MDS-MLD | 16 | 46, XY[20] | ND | 43.5 |
| 218 | 65 | M | 1 | 46,XY[10] | 1. *DNMT3A*: c.1903C>T; p.Arg635Trp; 12.4% | No | NA | NA | NA | NA | 58.7 |
| 217^§^ | 76 | M | 1 | 46,XY[20] | 1. *SF3B1:* c.2098A>G; p.Lys700Glu; 7.6% | No | NA | NA | NA | NA | 61.7 |
| 236^§◊^ | 69 | F | 1, 2 | 46,XX[20] | 1. *ASXL1*: c.1900_1922del;p.Glu635Argfs*15; 21% | No | NA | NA | NA | NA | 41.6 |
| 277^§^ | 77 | F | 1 | 46,XX[20] | 1. *TET2*: c.2539C>T; p.Gln847*; 41% | No | NA | NA | NA | NA | 55.4 |
| 242 | 58 | F | 4 | 46,XX[20] | 1. *TET2*: c.1771del; p.Gln591Serfs*10; 6.2% | No | NA | NA | NA | NA | 7.6 |
| 251 | 71 | F | 1, 3 | 46,XX[20] | 1. *ASXL1*: c.2423del; p.Pro808Leufs*10; 11.9% | No | NA | NA | NA | NA | 59.5 |
| 262^§^ | 83 | F | 1, 3 | 46,XX[20] | 1. *TET2*: c.1207C>T; p.Gln403*; 43% | No | NA | NA | NA | NA | 25.8 |
| 199^§^ | 84 | M | 1 | 46,XY[20] | 1. *ZRSR2*: c.827+2dup; p.?; 87% | No | NA | NA | NA | NA | 2.6 |
| 212^§^ | 72 | M | 1, 3 | 46,XY[20] | 1. *SF3B1*: c.2342A>G; p.Asp781Gly;26.7% | No | NA | NA | NA | NA | 63.8 |
| 285^§^ | 66 | M | 1, 3 | 46,XY[20] | 1. *TP53*: c.742C>T; p.Arg248Trp; 8% | No | NA | NA | NA | NA | 54.9 |
| 305 | 66 | M | 1 | 46,XY[20] | 1. *DNMT3A*: c.1555-3T>G; p.?; 17.2% | No | NA | NA | NA | NA | 15.4 |
| 194^§^ | 85 | M | 2, 3 | 46,XY[20] | 1. *DNMT3A*: c.1143_1158dup; p.Cys387Glufs*11; 23.8%; 2. *TET2*: c.687_688del; p.Leu230Valfs*23; 34.2%; 3. *TET2*: c.1692G>A; p.Trp564*; 44% | No | NA | NA | NA | NA | 52.7 |
| 306 | 19 | M | 3 | 46,XY[20] | 1. *ASXL1*: c.2638up; p.Thr880Asnfs*2; 11% | No | NA | NA | NA | NA | 64.4 |
| 275 | 80 | M | 4 | 46,XY,del(1)(p13p31.2)[17]/46,XY[3] | 1. *ASXL1*: c.1934dup; p.Gly646Trpfs*12; 12% | No | NA | NA | NA | NA | 0.6 |
| 202 | 57 | M | 4 | 46,XY,del(20)(q11.2q13.1)[4]/46,XY[16] | 1. *DNMT3A*: c.1668-2A>G; p.?; 17% | No | NA | NA | NA | NA | 51.6 |
| 220^§∆^ | 74 | F | 4 | del13q[6] | 1. *SF3B1:* c.2098A>G; p.Lys700Glu; 6.3% | No | NA | NA | NA | NA | 13 |
| 253 | 38 | M | 4 | 47,XY,+8[20] | None | Yes | MDS-EB1 | 10.5 | 47,XY,+8[20] | ND | 14.4 |
| 256 | 72 | F | 1, 3 | 45,X,-X[13]/46,XX[7] | None | No | NA | NA | NA | NA | 47.8 |
| 283 | 85 | M | 3 | 45,X,-Y[3]/46,XY[17] | None | No | NA | NA | NA | NA | 55.2 |
| 206 | 87 | M | 4 | 45,X,-Y[4]/46,XY[16] | None | No | NA | NA | NA | NA | 43.2 |
| 260 | 80 | M | 1, 3 | 45,X,-Y[7]/46,XY[13] | None | No | NA | NA | NA | NA | 40.4 |
| 240 | 79 | M | 1, 2 | 45,X,-Y[9]/46,XY[11] | None | No | NA | NA | NA | NA | 52.7 |
| 223 | 84 | M | 4 | 45,X,-Y[4]/46,XY[16] | None | No | NA | NA | NA | NA | 22.7 |
| 267 | 52 | F | 1, 3 | 46,XX,del(10)(q11.2q22)[20] | None | No | NA | NA | NA | NA | 23.2 |

MN, myeloid neoplasm; M, male; F, female; MDS, myelodysplastic syndrome; MDS-SLD, MDS with single lineage dysplasia; MDS-MLD, MDS with multilineage dysplasia; MDS-U, MDS, unclassifiable; MDS-EB1, MDS with excess blast-1. CMML-1, chronic myelomonocytic leukemia-1. ND, not done; NA, not applicable. Cytopenia category: 1. anemia; 2. neutropenia, 3. thrombocytopenia, 4. pancytopenia. ^§^CCUS cases harboring high-risk progression markers (CCUS-HR) of ≥20% VAF and/or non-DTA mutations.

^◊^CCUS cases with cardiovascular disease.

^∆^Although harboring del(13q) which was included in the WHO list of presumptive evidence for MDS, it did not provide definitive evidence of MDS in this case due to lack of persistent cytopenia (cytopenia resolved with subsiding of cytomegalovirus viremia).

Supplementary table 3. Performance of molecular marker variables in the diagnosis of MDS.

| **Variables** | **Sensitivity (95% CI)** | **Specificity (95% CI)** | **PPV (95% CI)** | **NPV (95% CI)** | **Youden's index** |
| --- | --- | --- | --- | --- | --- |
| ≥1 mutation | 84.7% (79.6-89.9%) | 77.6%( 70-85.2%) | 86.1% (81.1-91.0%) | 75.6% (67.9-83.3%) | 0.62 |
| ≥2 mutations* | 57.9% (50.9-64.9%) | 93.4% (88.7-98.1%) | 94.0% (89.7-98.3%) | 55.2% (48-62.6%) | 0.51 |
| ≥3 mutations* | 36.3% (29.5-43.2%) | 96.6% (93.2-99.8%) | 94.5(89.3-99.7%) | 48.1% (41.6-54.5%) | 0.33 |
| ≥4 mutations* | 21.1% (15.3-26.9%) | 100.0% (100-100%) | 100.0% (100-100%) | 43.6% (37.7-49.6%) | 0.21 |
| VAF ≥10% | 80.5% (74.9-86.2%) | 81.9% (74.9-88.9%) | 87.9% (83.1-92.8%) | 72% (64.3-79.6%) | 0.62 |
| VAF ≥20%* | 76.3% (70.3-82.4%) | 86.2% (79.9-92.5%) | 90.1% (85.4-94.7%) | 69% (61.4-76.5%) | 0.63 |
| VAF ≥30%* | 63.2% (56.3-70.0%) | 89.7% (84.1-95.2%) | 90.9% (86-95.8%) | 59.8% (52.5-67.1%) | 0.53 |
| ≥2 mutations and VAF ≥10%* | 56.8% (49.8-63.9%) | 94% (89.6-98.3%) | 93.9% (89.5-98.3%) | 57.1% (50-64%) | 0.51 |
| ≥2 mutations and VAF ≥20%* | 56.3% (49.3-63.4%) | 94% (89.6-98.3%) | 93.9% (89.5-98.3%) | 56.8% (49.8-63.8%) | 0.5 |
| ≥2 mutations and VAF >30%* | 54.2% (47.1-61.3%) | 94% (89.6-98.3%) | 93.6% (89.1-98.2%) | 55.6% (48.7-62.6%) | 0.48 |
| ≥3 mutations and any VAF ≥10%* | 35.8% (29.0-42.6%) | 96.6% (93.2-99.9%) | 94.4% (89.2-99.7%) | 47.9% (41.5-54.3%) | 0.32 |
| ≥3 mutations and any VAF ≥20%* | 35.8% (29.0-42.6%) | 96.6% (93.2-99.9%) | 94.4% (89.2-99.7%) | 47.9% (41.5-54.3%) | 0.32 |
| ≥3 mutations and any VAF ≥30%* | 31.0% (24.5-37.6%) | 96.6% (93.2-99.9%) | 93.7% (87.6-99.7%) | 46.1% (39.8-52.4%) | 0.28 |
| DTA-only mutations | 5.8% (2.5-9.1%) | 89.7% (84.1-95.2%) | 47.8% (27.4-68.2%) | 36.7% (31.1-42.4%) | -0.05 |
| Non-DTA mutations* | 78.4% (72.6-84.3%) | 87.9% (82-93.9%) | 91.4% (87.1-95.7%) | 71.3% (63.9-78.7%) | 0.66 |
| Epigenetic modifier (EM) mutations | 59% (52-65.9%) | 85.3% (78.9-91.8%) | 86.8% (81-92.7%) | 55.9% (48.6-63.2%) | 0.44 |
| Splicing factor (SF) mutations | 50.5% (43.4-57.6%) | 88.8% (83.1-94.5%) | 88.1% (82-94.2%) | 52.3% (45.3-59.3%) | 0.39 |
| Signaling and kinase pathway (SKP) mutations* | 11.1% (6.6-15.5%) | 100% (100-100%)% | 100% (100-100%) | 40.7% (35-46.4%) | 0.11 |
| Transcription factor (TF) mutations* | 20.5% (14.8-26.3%) | 100.0% (100-100%) | 100%(100-100%) | 43.4% (37.5-49.4%) | 0.21 |
| Tumor suppressor (TS) mutations* | 15.3% (10.2-20.4%) | 99.1% (97.5-100%) | 96.7% (90.2-100%) | 41.6% (35.9-47.5%) | 0.14 |

PPV, positive predictive value; NPV, negative predictive value; CI, confidence interval; VAF, variant allele fraction; DTA, *DNMT3A*, *TET2* and *ASXL1.* DTA-only mutations, mutations of the case restricted to DTA; non-DTA mutations, mutations of the case occurred in genes other than DTA, in the presence or absence of DTA mutations. *Variable with ≥90% PPV or specificity.

Supplementary table 4. Cox proportional hazard ratio analysis of MN progression risk variables in 116 noMN patients.

| **Risk variables** | **Hazard Ratio** | **95% CI** | ***p* value** |
| --- | --- | --- | --- |
| Univariate analysis |  |  |  |
| Age, ≥70 vs. <70 years | 4.8 | 1.27-18.1 | 0.02* |
| Gender, M/F | 6.6 | 0.85-51.9 | 0.07 |
| VAF ≥ 20%^a^ | 59.5 | 7.6-466.4 | 0.0001* |
| Non-DTA mutations ^a^ | 35.7 | 7.7-166.1 | <0.0001* |
| ≥ 2 mutations ^a^ | 16.0 | 4.9-52.8 | <0.0001* |
| Cytogenetic abnormalities ^a^ | 0.8 | 0.1-6.0 | 0.8 |
| Hb, <10 vs. ≥10 g/dL | 1.1 | 0.3-3.5 | 0.91 |
| ANC, <1.8 vs. ≥1.8 x10^9^/L | 2.2 | 0.7-7.6 | 0.21 |
| Platelet count, <100 vs. ≥100 x10^9^/L | 0.6 | 0.2-1.9 | 0.34 |
| Multivariate analysis |  |  |  |
| Age, ≥70 vs. <70 years | 0.1 | 0.001-2.2 | 0.15 |
| Gender, M/F | 11.3 | 0.5-271.3 | 0.14 |
| VAF ≥20% ^a^ | 44.8 | 2.5-811.8 | 0.01* |
| Non-DTA mutations ^a^ | 13.1 | 1.1-162.5 | 0.046* |
| ≥2 mutations ^a^ | 1.2 | 0.3-4.7 | 0.8 |

MN, myeloid neoplasm; M, male; F, female; VAF, variant allele fraction; DTA, *DNMT3A*, *TET2*, or *ASXL1* mutations, M/F, male/female. Hb, hemoglobin; ANC, absolute neutrophil count.

^a^ Present vs. absent.

*Statistically significant with *p* <0.05.

Supplementary table 5. Clinical and hematological features of low-risk MDS (MDS-LR) and high-risk CCUS (CCUS-HR).

|  | **MDS-LR (n=72)** | **CCUS-HR (n=19)** | ***p* value** |
| --- | --- | --- | --- |
| Age, mean (SD, range), years | 72.5 (8.9, 48.7-87.1) | 75.7 (5.9, 66.4-85.4) | 0.14 |
| Gender (male/female) | 54/22 | 14/5 | 1.0 |
| Hb, mean (SD, range), g/dL | 9.6 (1.8, 6.5-14.6) | 9.6 (2.0, 6.7-14.2) | 0.9 |
| ANC, mean (SD, range), x10^9^/L | 2.2 (2.2, 0.2-15.5) | 2.2 (2.2, 0.06-8.7) | 1.0 |
| Platelet count, mean (SD, range), x10^9^/L | 172 (127, 29-589) | 121 (64, 30-223) | 0.09 |

MDS, myelodysplastic syndromes; CCUS, clonal cytopenia with undetermined significance; Hb, hemoglobin; ANC, absolute neutrophil count.
